# Investigations of Structural Requirements for BRD4 Inhibitors through Ligand- and Structure-Based 3D QSAR Approaches

**DOI:** 10.3390/molecules23071527

**Published:** 2018-06-25

**Authors:** Adeena Tahir, Rima D. Alharthy, Saadia Naseem, Natasha Mahmood, Mahmood Ahmed, Khuram Shahzad, Malik Nadeem Akhtar, Abdul Hameed, Irfan Sadiq, Haq Nawaz, Muhammad Muddassar

**Affiliations:** 1 Department of Biosciences, COMSATS University Islamabad, Park Road, Islamabad 45550, Pakistan; adeena.tahir@yahoo.com (A.T.); saadia.naseem@comsats.edu.pk (S.N.); natasha.mahmood@gmail.com (N.M.); khuramsb@gmail.com (K.S.); nadeemakhtar@comsats.edu.pk (M.N.A.); irfan_sadiq@comsats.edu.pk (I.S.); 2 Department of Chemistry, Science and Arts College, Rabigh Campus, King Abdulaziz University, Jeddah 21577, Saudi Arabia; iaaalharte@kau.edu.sa; 3 Institute of Chemistry, University of the Punjab, Lahore 54590, Pakistan; mahmoodresearchscholar@gmail.com; 4 H.E.J. Research Institute of Chemistry, International Center for Chemical and Biological Sciences, University of Karachi, Karachi 75270, Pakistan; abdul_hameed8@hotmail.com; 5 Department of Chemistry, University of Agriculture, Faisalabad 38040, Pakistan; haqchemist@yahoo.com

**Keywords:** BRD4 protein inhibitors, molecular docking, 3D-QSAR, CoMFA, CoMSIA

## Abstract

The bromodomain containing protein 4 (BRD4) recognizes acetylated histone proteins and plays numerous roles in the progression of a wide range of cancers, due to which it is under intense investigation as a novel anti-cancer drug target. In the present study, we performed three-dimensional quantitative structure activity relationship (3D-QSAR) molecular modeling on a series of 60 inhibitors of BRD4 protein using ligand- and structure-based alignment and different partial charges assignment methods by employing comparative molecular field analysis (CoMFA) and comparative molecular similarity indices analysis (CoMSIA) approaches. The developed models were validated using various statistical methods, including non-cross validated correlation coefficient (r^2^), leave-one-out (LOO) cross validated correlation coefficient (q^2^), bootstrapping, and Fisher’s randomization test. The highly reliable and predictive CoMFA (q^2^ = 0.569, r^2^ = 0.979) and CoMSIA (q^2^ = 0.500, r^2^ = 0.982) models were obtained from a structure-based 3D-QSAR approach using Merck molecular force field (MMFF94). The best models demonstrate that electrostatic and steric fields play an important role in the biological activities of these compounds. Hence, based on the contour maps information, new compounds were designed, and their binding modes were elucidated in BRD4 protein’s active site. Further, the activities and physicochemical properties of the designed molecules were also predicted using the best 3D-QSAR models. We believe that predicted models will help us to understand the structural requirements of BRD4 protein inhibitors that belong to quinolinone and quinazolinone classes for the designing of better active compounds.

## 1. Introduction

The bromodomain containing protein 4 (BRD4) is a key therapeutic target for Bromodomain and extra-terminal domain (BET) inhibitors, a group of pharmaceutical drugs that have recently gone under the clinical trials [1,2]. BRD4 plays a vital role in the expression of “tumor driving” oncogenes, as shown in myeloid leukemia, multiple myeloma, and basal-like breast cancer [3,4]. It has been observed that the BRD4 protein regulates the expression of the *MYC* transcription factor (a master regulator) in cellular proliferation of numerous cancerous pathways [5]. The decreased amount of BRD4 expression results in reduced activity of *MYC* oncogene, which is a potential therapeutic target in different cancer studies [5,6,7]. The inhibition of this protein is of significant interest for the usage of BET inhibitors as therapeutic interventions for the treatment of various cancer types, inflammatory reactions, and cardiovascular diseases [8]. 

The BRD4 protein interacts with different classes of compounds based on their chemical structures. These classes of compounds are known as thienotriazolodiazepine (JQ1, the very first BRD4 inhibitors reported in 2010), tetra hydro-quinoline, 3,5-dimethylisoxzole, and 2-thiazolidinone derivatives [9]. Several other known inhibitory molecules, such as MS417, AZD5153, ZL0420, and ZL0454, interact with the BRD4 protein to interrupt its cellular activities. The interaction with BRD4-inhibitor MS417 causes downregulation of NF-κB transcriptional activity, as observed in HIV- associated renal disease [10]. In another study, MS417 has been used in the treatment of colorectal cancer due to its inhibitory effects [11]. The compound AZD5153 is involved in the treatment of thyroid carcinoma, which activates apoptosis and caspase activities in the cell [12]. The latter two compounds, ZL0420 and ZL0454, have been recently identified for the treatment of airway inflammation in mouse models using molecular docking studies [13].

In the current study, we investigated structural requirements to design better active inhibitors of BRD4 protein from quinolinone and quinazolinone classes. We employed comparative molecular field analysis (CoMFA) [14] and comparative molecular similarity indices analysis (CoMSIA) [15] methods to drive three-dimensional quantitative structure activity relationship (3D-QSAR) models along with molecular docking simulations. In this case, structural properties were correlated with the biological activities of small molecules, which were further evaluated using different statistical methods. In CoMFA modeling, steric and electrostatic fields of molecules were correlated with their biological activities [16], while in CoMSIA modeling, hydrophobic, hydrogen bond donor and acceptor fields, along with steric and electrostatic fields were correlated with activities [17]. Afterwards, key structural features were identified based on the best generated model, and then, new molecules were designed to explore better active compounds.

## 2. Results and Discussion

### 2.1. Statistical Analyses of CoMFA and CoMSIA Models

**Figure 1:**
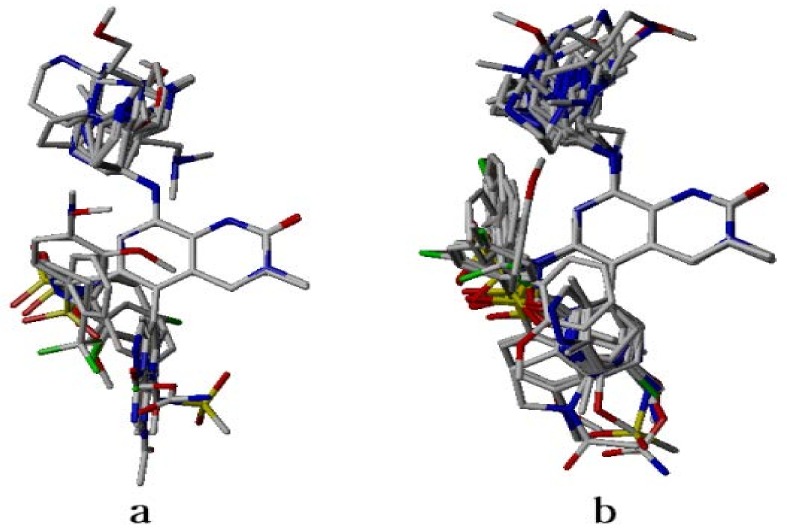
Alignment of dataset compounds (**a**) Ligand-based alignment of the conformers obtained from omega software; (**b**) Structure-based alignment of docked compounds.

Different CoMFA- and CoMSIA-based 3D-QSAR models were generated using partial least square method (PLS) by correlating biological activities of BRD4 inhibitors in a training dataset with their field descriptors. There are several factors that affect the quality of the developed CoMFA and CoMSIA models [18]. However, the alignment of the dataset molecule and the charges assigned to them are the two major factors that affect the predictability of the generated models [19]. In this study, alignment methods, such as ligand- and receptor-based, as shown in Figure 1, along with partial charges methods like Merck molecular force field (MMFF94), Gasteiger Huckle (GH), and Gasteiger Marsilli (GM) were evaluated to obtain the best predictive CoMFA and CoMSIA models [20]. The structure-based conformation alignment method with MMFF94 charges yielded the best models. The leave-one-out (LOO) cross validated correlation coefficient (q^2^) value with both steric and electrostatic fields in CoMFA is 0.569, along with optimum number of components (ONC) = 5, standard error of estimate (SEE) = 0.102, non-cross validated coefficient (r^2^_ncv_) = 0.979, *F*-value = 336.72, and r^2^_pred_ = 0.816, as given in Table 1. The model shares 47% contribution of steric and 53% electrostatic fields. In CoMFA modeling, different charges did not influence the predictive quality of the models (data shown in Supplementary File, Tables S1 and S2). Similarly, models generated using ligand-based alignment and MMFF94 charge method yielded q^2^ = 0.399 value with both steric and electrostatic fields in CoMFA with ONC = 2 (see Table 1). The effects of different charges on the statistical models in ligand-based alignment method are given in the Supplementary File, Tables S3 and S4.

For CoMSIA models using the MMFF94 charge method with structure-based alignment yielded q^2^ = 0.500 with ONC = 6, SEE = 0.094, *F*-value = 396.442, r^2^_ncv_ = 0.982, and r^2^_pred_ =0.834 (Table 1). Different field contributions, such as steric, electrostatic, hydrophobic, hydrogen bond donor, and hydrogen bond acceptor were 0.130, 0.345, 0.254, 0.127, and 0.144, respectively, for this model. The results showed that both electrostatic and hydrophobic fields played a substantial role in the CoMSIA model, while steric and electrostatic fields played an important role in the CoMFA models with 0.470 and 0.530 contribution, respectively. CoMSIA models generated based on GH and GM charges were not statistically significant. The reasons for the better performance of one charge method over the other in a CoMFA/CoMSIA model is unknown as of yet, as the prior literature provides conflicting results of different charge-based methods.

**Figure 2:**
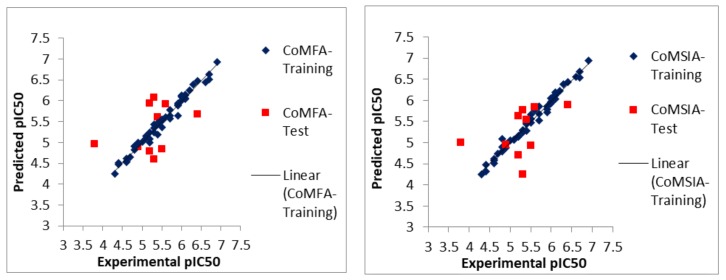
Experimental vs predicted biological activities (pIC_50_) values from best structure-based three-dimensional quantitative structure activity relationship (3D-QSAR) models.

Further, receptor-based CoMFA and CoMSIA models were used to predict the activities of the training and test dataset compounds. The experimental and predicted activities are shown in Figure 2. The scatter plots depict that all points are situated around the diagonal lines, and there is no obvious deviated point present on them. The higher r^2^_pred_ values for both CoMFA and CoMSIA models for external validation confirm that the models are of good quality. Similarly, internal validations like r^2^_ncv_, *F*-values, and r^2^_bs_ (bootstrapping) values show that the models are quite stable and accurate which can be further used to design better active compounds. As the statistical significance of a model is affected by individual fields that are totally independent from each other, so the best models were selected to design new compounds with improved activities. The designing was performed with the help of indicated regions in 3D color contour maps [21].

### 2.2. CoMFA Contour Maps

**Figure 3:**
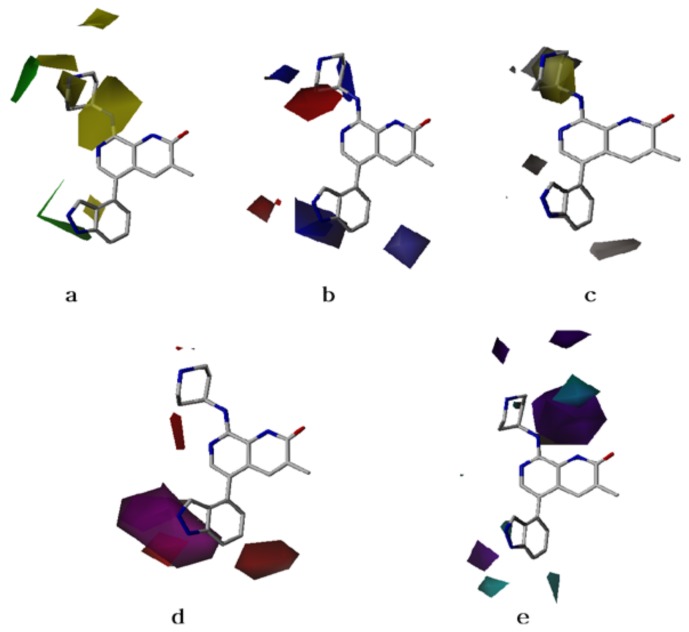
Contour maps of structure-based model on the most active compound (**42**) as template (sticks). (**a**) CoMFA steric field contour maps; (**b**) CoMFA electrostatic fields contour maps; (**c**) CoMSIA hydrophobic field contour maps; (**d**) CoMSIA hydrogen bond acceptor fields contour maps; and (**e**) CoMSIA hydrogen bond donor fields contour maps.

Contour maps are the graphical interpretation of the generated statistical models that provide information about the compounds in a 3D space where substitution with functional groups may alter the biological activities of the compounds. The CoMFA contour maps of steric and electrostatic fields from the best CoMFA model of a highly active compound are shown in Figure 3a,b. In Figure 3a, the green contour represents the favored area of bulky group substitution, while the yellow contour indicates that the lighter group substitution will be favorable to enhance the biological activities of the compounds. In Figure 3b, the blue contour indicates the electron-donating group, and the red contour represents that the electron-withdrawing group will be favorable to improve the activity.

The obtained 3D-QSAR models were used to explain the structure activity relationship of the dataset compounds (see “material and methods” section). The bulky group substitution at the green contour near the R3 position of the highly active compound, as shown in Figure 3a, becomes the R1 position for the compounds (**1–13**); however, the lighter group at the yellow contour located towards N-methyl piperdine will enhance the biological activity of the compound. These contours may explain why compounds, such as **4–11**, having bulkier groups with substituted phenyl rings, are more active than compounds **1–3**, having lighter groups. Similarly, compounds **14**, **17**, and **28**, having lighter groups at R2 position, are more active than compounds **32–38**, which possess the bulkier groups at this position.

In electrostatic contours, as shown in Figure 3b, the presence of the large blue contour near the R3 position of 3-methyl-1,7-naphthyridin-2(1H)-one suggests that electron-donating groups will likely enhance the biological activity of the designed compounds. Compounds **55**, **57–59**, which have pyridine rings with different electron-donating substituent groups, are more active than compounds **51**, **54**, **56**, and **60** possessing only heterocyclic rings. Similarly, the large red contour near the R2 position suggests that an electron-withdrawing group is desirable for improving the activity. Therefore, compounds such as **14**, **15**, **28**, and **30** are less active than **22** and **34** because they have an electron-donating group near the R2 position.

### 2.3. CoMSIA Contour Maps

CoMSIA contour maps of the steric and electrostatic fields were similar to CoMFA models. The contour maps of the hydrophobic, hydrogen bond donor, and acceptor fields are shown in Figure 3c–e. The hydrophobic field contour reveals that the big yellow contour at the R2 position is hydrophobic in nature, while the white contour at R3 is hydrophilic in nature to enhance the activity (see Figure 3c). In the dataset, we can observe that compounds **34**, **37**, and **38**, having hydrophobic groups at the R2 position, are exhibiting higher activities compared to other compounds having hydrophilic groups at this position. 

Hydrogen bond acceptor and donor fields of the CoMSIA model are shown in Figure 3d,e, respectively. The magenta contour in Figure 3d indicates the region where hydrogen bond-accepting substituents enhance the inhibitory activity, while the red contour specifies the regions where hydrogen bond-accepting moiety may deteriorate the biological activity of the compounds. Further, the hydrogen bond acceptor contour in Figure 3d corresponds to the electron-donating group in the electrostatic field contour map of Figure 3b. Similarly, the cyan contour at the R2 position in Figure 3e favors the hydrogen bond-donating moieties for enhancing the activity, whereas the purple contour disfavors the areas for such moieties to increase the activity of the compounds. This hydrogen bond-donating contour is near to the electropositive contour in the electrostatic contour of Figure 3b. Hence, from the information in these contour maps, it is clear that the hydrogen bond acceptor/donor contours nearly coincide to the electron-donating and -accepting favored contours for enhancing the biological activities of the compounds. In these figures, the contribution of the hydrogen bond acceptor and donor regions is 70% for favorable and 30% for non-favorable areas in terms of enhancing the activity.

### 2.4. Analysis of Structure-Based Generated Conformations

**Figure 4:**
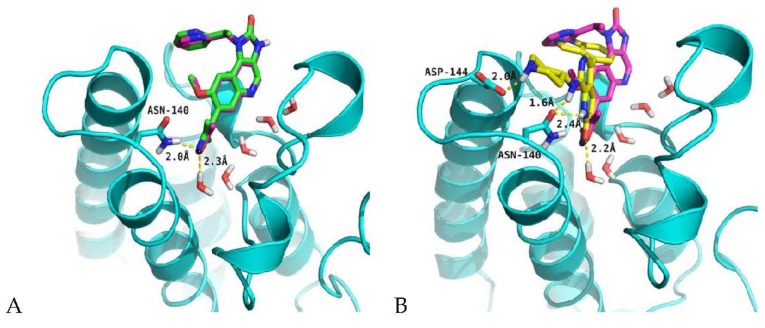
Docking pose of the ligands in the active site of the BRD4 protein (cyan) along with water molecules (red and white sticks). (**A**) Superposition of best pose of the co-crystal ligand (green) on the bound ligand (magenta) after redocking experiments; (**B**) Superposition of the docked pose of the most active compound (yellow) on the co-crystal ligand (magenta). The yellow dotted lines represent hydrogen bonds.

Molecular docking studies were performed to assess the performance of the glide docking score. The co-crystal ligand (I-BET151) in human BRD4 was first removed from the protein structure and then sketched, minimized, and redocked at the binding site. The best binding mode, with a glide score of -6.76 kcal/mol, was superimposed on the co-crystal ligand as shown in Figure 4A. The docked pose exhibited a similar interaction pattern as was present in the crystal structure [22]. The ASN140 residue of the BRD4 protein and one water molecule were making the hydrogen-bonding interaction with nitrogen and oxygen atoms of the isoxazole moiety, respectively. The distances between these interacting atoms was 2 Å and 2.3 Å. The presence of water molecules at the bottom of the active site is essential, because co-crystal ligand interacts with TYR97 [23] indirectly by hydrogen bonding through a water molecule. The water molecules also prevent the direct contact of acetylated lysine of histone at the bottom of the active site [24]. Keeping this in mind, water molecules were kept in the binding site while docking all dataset compounds. The binding pose of the best active compound (**42**) with the co-crystal ligand is shown in Figure 4B. The best pose yielded a −6.70 kcal/mol glide score with side chain hydrogen bonding interactions. The hydrogen-bonding pattern is similar to the co-crystal ligand, in which ASN140 and the water molecules present at the bottom are interacting with carbonyl oxygen of 3-methyl-1,7-naphthyridin-2(1H)-one scaffold. Similarly, an extra hydrogen bond is also present in the form of a salt bridge between the ASP144 and N-H of the pipridine moiety of the compound. 

### 2.5. Designing of New Compounds and Their Physicochemical Properties’ Calculations

Using the best CoMFA and CoMSIA models generated by receptor-based modeling, ten new BRD4 inhibitors were designed using contour map information by attaching different substituents at various positions of 3-methyl-1,7-naphthyridin-2(1H)-one. The physicochemical properties, as shown in Table 2, were predicted using QikProp software. We have found that most of the newly designed compounds followed the Lipniski drug-like rules with almost one rule violation. The predicted octanol/water partition coefficient (QPlogPo/w) values between 1.232 and 4.433, HERG K+ channels (QPlogHERG) blocking IC_50_ values between −6.894 and −5.899, caco-2 cell permeability (QPPCaco) values between 13.145 and 195.227, brain/blood partition coefficient (QPlogBB) values between −3.049 and −1.354, and human serum albumin binding (QPkhsa) values between −0.067 and 0.722 are within the acceptable ranges for 95% oral drugs as described in [25]. These physicochemical properties (e.g., QPlogPo/w and QPlogHERG) within the recommended ranges ensure the smooth distribution of drug functioning and prevention against sudden risks of cardiac arrest, respectively.

### 2.6. Biological Activities Prediction of Newly Designed Compounds

**Figure 5:**
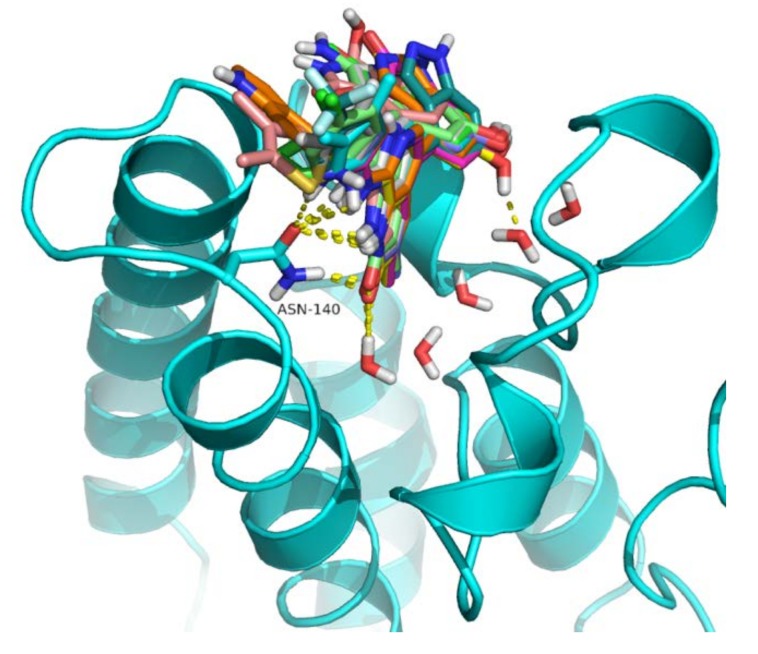
Docking pose of the newly designed compounds in the active site of the BRD4 protein (cyan) along with water molecules (red and white sticks). The yellow dotted lines represent hydrogen bonds.

The biological activities of newly designed compounds listed in Table 3 were predicted using the best CoMFA and CoMSIA models. Before the activities’ prediction, molecular alignment of the newly designed molecules was achieved using docking simulations. The docking studies showed that these compounds possessed similar binding modes, as shown in Figure 5. The interaction pattern is similar to that of the other compounds present in the dataset. All of the designed compounds make H-bonding with ASN140 and indirectly with TYR97 through water molecules that lie at the bottom of the active site. The docking scores and predicted activities are reported in Table 3. The predicted activities of these newly designed compounds are better than most of training dataset compounds. These results prove that generated 3D-QSAR models with significant predictive ability could be used for structural optimization of the newly designed compounds. 

## 3. Material and Methods

### 3.1. Biological Data Collection

The set of 60 chemically diverse inhibitors of BRD4 protein, having similar chemical scaffold, were collected from the literature. The compounds used as racemic mixtures for biological activities [26,27,28] were not selected for the final data set. The chemical structures of the compounds and biological data along with their pIC50 values are listed in Table 4. Further, for model building and validation, the selected compounds were divided into training and test datasets using a random selection method [29]. In total, 50 compounds were included in training, while 10 were added in test datasets to evaluate the predictability of the generated models. 

### 3.2. Dataset Compounds Modeling and Alignment

The 3D structures of the inhibitors were sketched in the SYBYL-X 2.1.1 molecular modeling package. The omega tool from OpenEye Scientific Software [30] was used for conformational search of each molecule. Finally, the lowest energy conformer was selected from all the resulting structural conformations. The partial charges were calculated using different methods including GH, GM, and MMFF94 charges [31,32]. Because the alignment of molecules is believed to be the most crucial and important requirement for a 3D-QSAR model’s robustness and predictability, the alignments were performed based on common substructures of the template molecule and all the other compounds present in the dataset. Due to the highest inhibitory activity, compound **42** was considered to be a template molecule. For ligand-based modeling, alignment was obtained after superposition of the lowest energy conformation of each molecule obtained from the omega tool on the compound **42**. However, for structure-based modeling, the alignment was performed on the conformations obtained after the molecular docking simulations. 

### 3.3. CoMFA and CoMSIA Fields Calculations

For CoMFA steric and electrostatic field calculations, a 3D cubic lattice with 2.0 Å grid spacing in the X, Y, and Z coordinates was generated using a default value in SYBYL. The energy cut-off was fixed to ±30 kcal/mol to get rid of high energy values. For the steric probe, an sp3-hybridized carbon was taken while +1.0 charge was selected as an electrostatic probe atom. The five fields of CoMSIA including steric, electrostatic, hydrophobic, and hydrogen bond donor and acceptor were also calculated using a probe atom with a radius of 1.0 Å. The default value of 0.3 for the attenuation factor (α) was used, which calculates the standard distance dependent similarity indices. Similarly, an indices calculation was performed by the following equation 1. All the numerical calculations were executed in the same way as for the CoMFA analysis [31,33,34].

(1) $$\;A_{F,k}^{q}\left(j\right)=\sum^{}{ɷ}_{probe,k}{ɷ}_{ik}e^{-ar^{2}}iq$$

In the above equation, *A^q^* means the similarity index of point *q*; *k* denotes the physiochemical properties of steric and electrostatic descriptors; *ω_probe,k_* represents the probe atom; *i* denotes the summation index of molecule *j*, while *ω_ik_* is the observed value k of a specific property of the atom *I*, and r is the atomic radius [35].

### 3.4. Partial Least Squares (PLS) Regression Analyses and Validations of the Models

PLS regression analysis remained very useful for 3D-QSAR models in which CoMFA and CoMSIA descriptors were used as dependent variables and biological activities are taken as independent variables [36]. For selecting the best model, which probably has the maximum predictability, the cross-validation analysis using the LOO method was used, which can be defined by the following Equation (2):

(2) $$\;q^{2}=1-\;\frac{\sum_{y}\;\;{\left(y_{pred\;}-y_{obs\;}\right)}^{2}}{\sum_{y}{\left(y_{obs\;}-y_{mean\;}\right)}^{2}}$$

where each value represents the predicted (*y_pred_*), experimental (*y_obs_*), and mean (*y_mean_*) values of the target property. By using ONC, non-cross validated analysis was performed without column filtering. Along with cross and non-cross validated r^2^, SEE values were calculated using SYBYL. For further validation of generated models, bootstrapping analyses were performed for 100 runs to evaluate the effectiveness of the derived models. Predictive r^2^, based on the test set molecules, was used to express the predictive ability of the generated models. The predictive r^2^ is defined by the following Equation (3):

(3) $$r_{pred}^{2}=\frac{\left(SD-PRESS\right)}{SD}$$

In the above equation, SD is the sum of the squared deviations between the biological activities of the test set and the mean activities of the training set molecules. Whereas *PRESS* is the sum of the squared deviations between the observed and the predicted activities of the test molecules.

### 3.5. Preparation of Ligands and Protein Structure

For the molecular docking studies, dataset compounds were prepared using the ligprep tool embedded in Schrodinger software (www.schrodinger.com). The possible ionization states and stereoisomers were generated by keeping a maximum of 32 conformations of each molecule using an OPLS2005 forcefield. The BRD4 protein crystal structure [37] (PDB ID: 3zyu), having the resolution of 1.5 Å, was retrieved from the protein data bank (www.rcsb.org). Missing hydrogen atoms were added and other unnecessary crystal ligands such as 1,2-ethanediol and buffer reagents were removed. The water molecules were also removed except the four present at the bottom of the active site. Finally, restrained minimization was performed using the OPLS2005 forcefield to remove steric clashes. Meanwhile, conformation of entire protein-ligand complex was allowed to deviate 0.30 Å root mean square deviation from its native structure. 

### 3.6. Molecular Docking Protocol

To predict the plausible binding modes of the compounds and get the structure-based alignment for the CoMFA and CoMSIA modeling, the prepared crystal structure of the BRD4 protein was employed for receptor grid generation using Schrodinger software. During the grid box generation, the active site was considered where the co-crystalized ligand (GSK1210151A) is present in the protein. The X, Y, and Z coordinates at 0.76, −8.19, and 22.37 were defined with 10 Å length in each dimension. The hydrogen atoms of the hydroxyl and thiol groups of amino acids present in the active site were permitted to rotate during the molecular docking simulations. No other torsional or positional restraint was applied except the hydrogen bond formation with the ASN140 residue. During the docking simulation, softening potential for the non-polar parts of the receptor was applied by adjusting the scaling factor of van der Waal’s radii to 0.80 with a cut-off value of 0.15 along with other parameters’ default settings. Among the all docking poses of the docked compounds, only the top five poses of each compound were subjected to minimization. Eventually, the best pose was selected based on the highest glide score and best binding mode using standard precision (sp) mode in glide [38].

## 4. Conclusions

The BRD4 protein plays various roles in the progression of different types of cancers, which makes it an attractive drug target. In this study, we performed a 3D-QSAR modeling using CoMFA and CoMSIA approaches on series of 60 BRD4 protein inhibitor molecules containing quinolinone and quinazolinone as central scaffolds. Several statistical models were generated using the lowest energy- and structure-based bioactive conformations using different charge methods. The best predictive models were generated using different molecular alignment and charges-based methods. The docking-based alignment method with MMF94 charges yielded the best CoMFA and CoMSIA models. The contour maps suggest that the bulky electron-donating group near the R3 position and the lighter electron-withdrawing groups near the R2 position will help to enhance the biological activities of this series of compounds. Based on contour maps information of the best models, ten new compounds were designed, and their biological activities were predicted. Their binding interactions with BRD4 protein were also assessed using docking simulations. Finally, the predicted pharmacokinetic properties showed that most of the designed molecules are in the acceptable ranges of the majority of the oral drug values. Hence, we believe that developed 3D-QSAR models could be useful for the development of more potent BRD4 inhibitors.

## Figures and Tables

**Table 1:** Statistical Results of Structure- and Ligand-Based Models.

MMFF94 Charges	Structure-Based Model		Ligand-Based Model	
	50 Compounds			
Parameters	CoMFA	CoMSIA	CoMFA	CoMSIA
**ONC**	5	6	2	6
**q^2^_(loo)_**	0.569	0.500	0.399	0.403
**r^2^_(ncv)_**	0.979	0.982	0.873	0.873
**SEE**	0.102	0.094	0.251	0.251
**F**	336.723	396.442	49.120	49.133
**Pred-r^2^**	0.816	0.834	0.762	0.769
**Steric (S)**	0.470	0.130	0.474	0.120
**Electrostatic (E)**	0.530	0.345	0.526	0.328
**Hydrophobic (H)**	-	0.254	-	0.213
**Donor (D)**	-	0.127	-	0.166
**Acceptor (A)**	-	0.144	-	0.173
**r^2^_bs_**	0.988	0.988	0.915	0.927
**SD_bs_**	0.004	0.005	0.066	0.058

Merck molecular force field (MMFF94); comparative molecular field analysis (CoMFA); comparative molecular similarity indices analysis (CoMSIA); ONC = optimal number of components; q^2^ = cross-validated correlation coefficient; r^2^ = determination coefficient; r^2^_ncv_ = non-cross validated coefficient; SEE = standard error of estimate; F = Fischer’s *F*-value; Pred-r^2^ = predictive r^2^; r^2^_bs_ = r^2^ obtained after bootstrapping; and SD_bs_ = bootstrapping standard deviation.

**Table 2:** Predicted physicochemical/pharmacokinetic properties of the newly designed compounds.

Comp	MW	HBD	HBA	QPlogPo/w	QPlogHERG	QPCaco2	QPlogBB	QPlogKhsa
**1**	405.41	6	7	1.232	−5.899	15.239	−2.972	−0.046
**2**	416.43	5	6	2.732	−6.434	76.171	−2.227	0.358
**3**	404.38	6	6	1.606	−6.432	23.449	−2.752	−0.022
**4**	403.39	7	5	1.373	−6.352	13.145	−3.049	−0.067
**5**	432.49	4	8	2.371	−6.23	62.067	−2.187	0.246
**6**	432.49	5	5	3.191	−6.294	80.298	−2.084	0.478
**7**	451.31	4	4	4.433	−6.894	195.227	−1.354	0.717
**8**	420.47	3	5	3.653	−6.669	107.71	−1.933	0.722
**9**	431.88	6	5	2.937	−6.857	44.197	−2.357	0.332
**10**	451.41	4	5	3.546	−6.692	75.418	−1.887	0.54

MW = molecular weight, HBD = hydrogen bond donor, HBA = hydrogen bond acceptor, QPlogPo/w = octanol/water partition coefficient (recommended rage −2.0 to 6.5), QPlogHERG = blockage of HERG K+ channels (recommended range <−5), QPCaco2 = Caco2 cell permeability (recommended range <25 poor, >500 great), QPlogBB = brain/blood partition coefficient (recommended range −3.0 to 1.2), and QPlogKhsa = binding to human serum albumin (recommended range −1.5 to 1.5).

**Table 3:** Newly designed compounds structures with their docking scores and predicted biological activities.

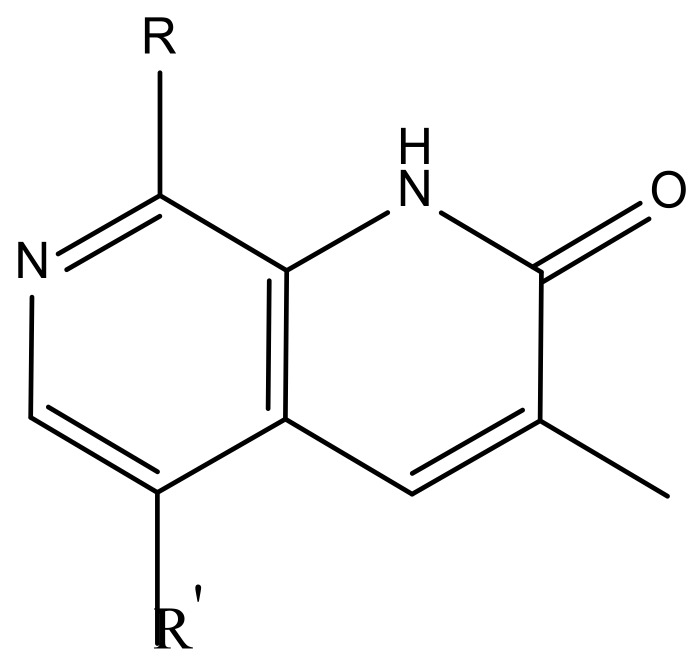

	Substituents		Glide-Score	Predicted pIC_50_	
No.	R	R′		CoMFA Model	CoMSIA Model
1	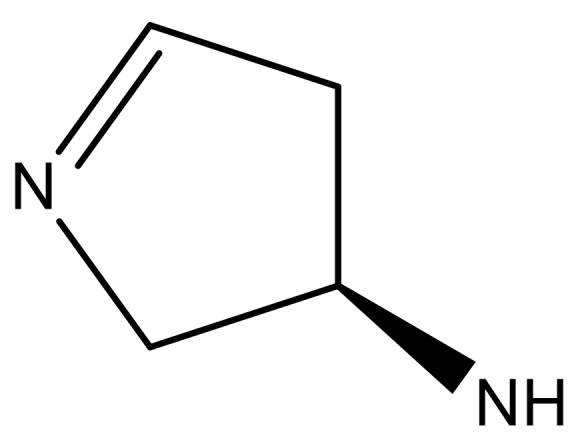	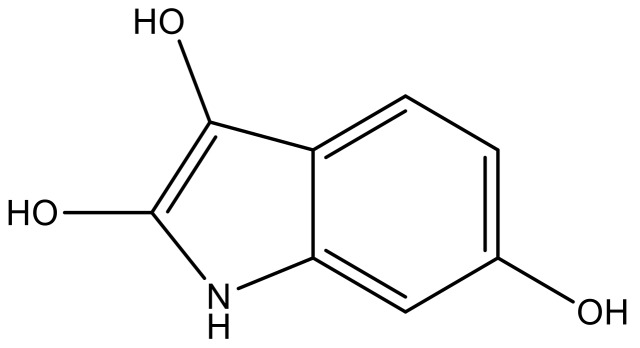	−7.348	7.675	6.757
2	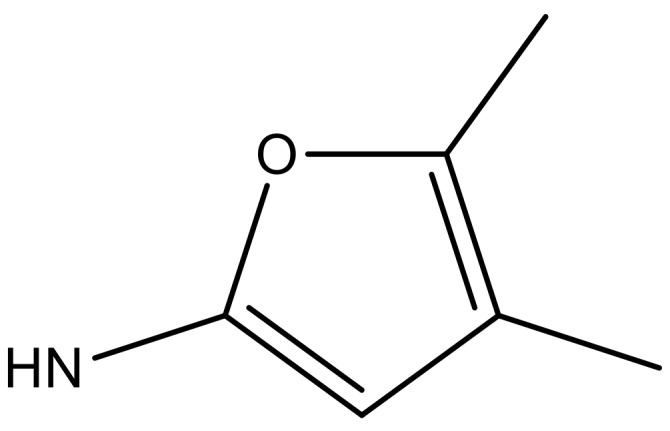	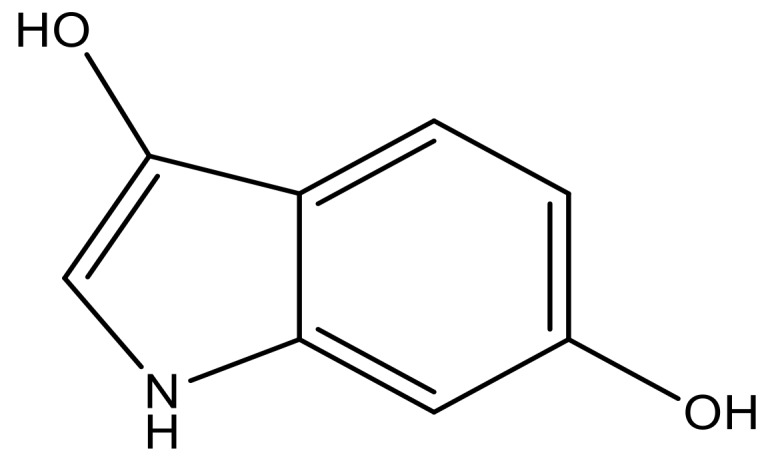	−7.140	7.293	6.523
3	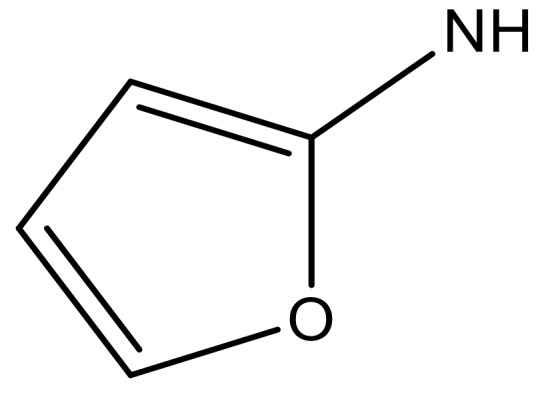	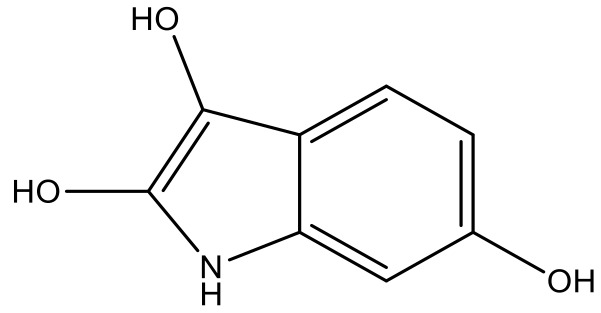	−7.775	7.116	6.219
4	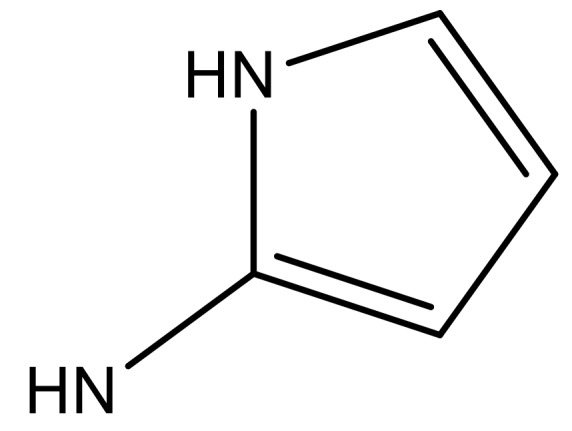	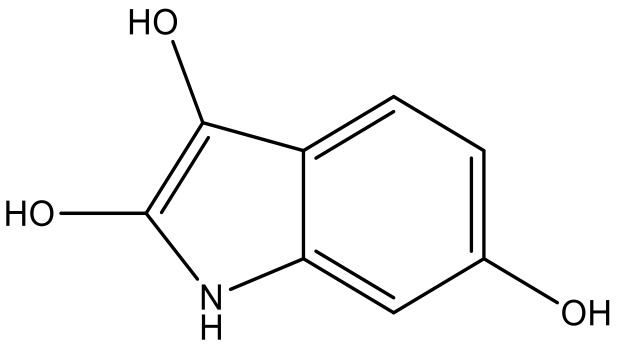	−7.010	7.113	6.074
5	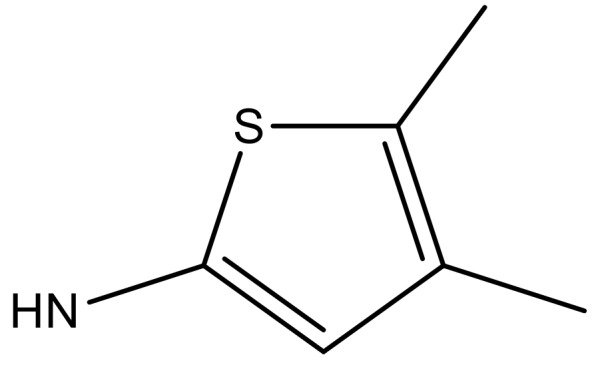	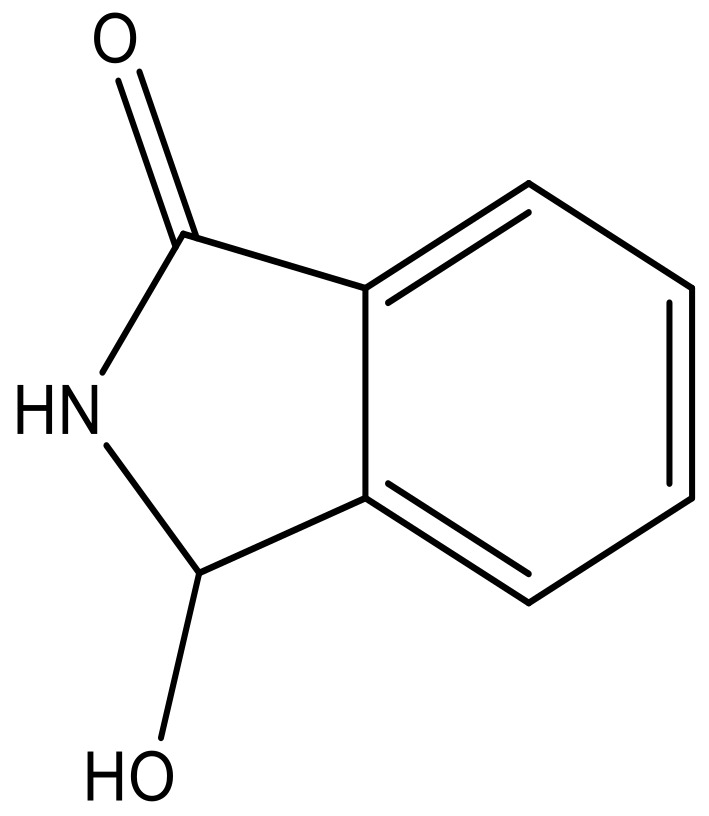	−5.953	6.922	6.255
6	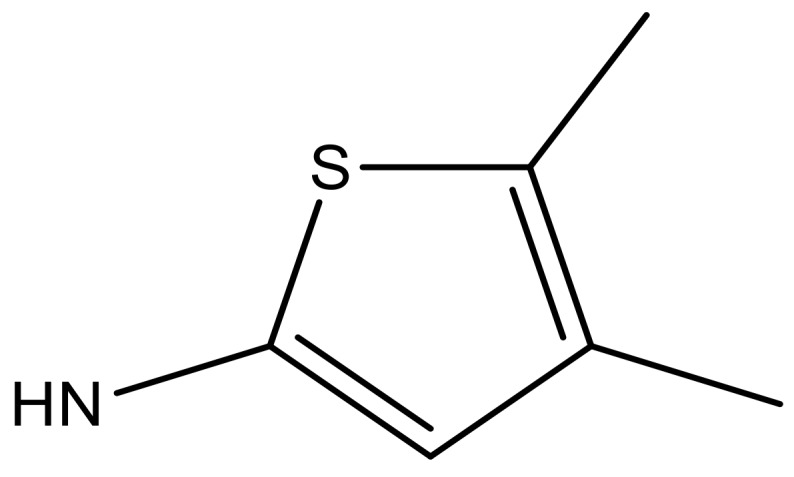	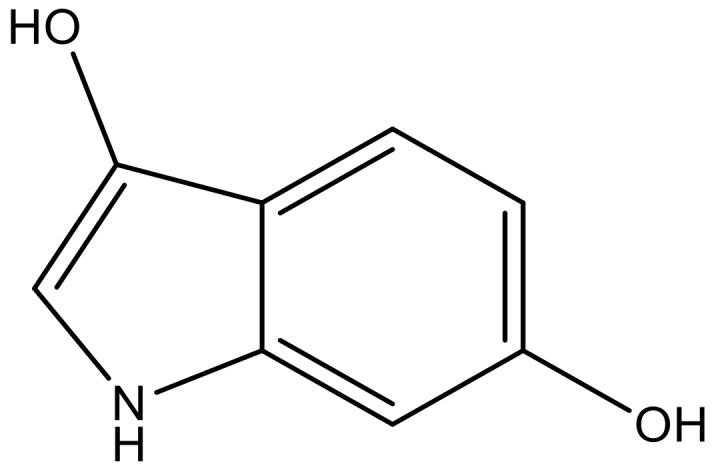	−6.691	6.740	6.614
7	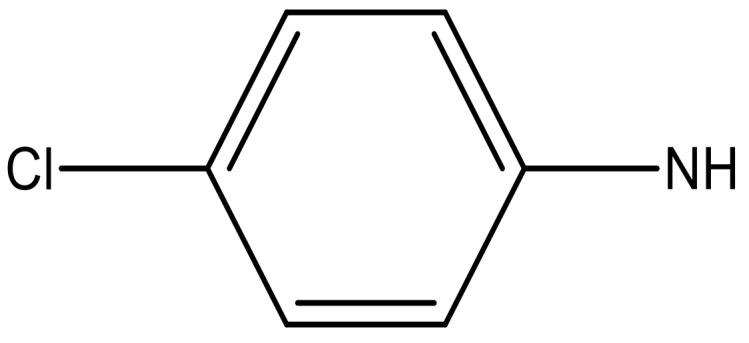	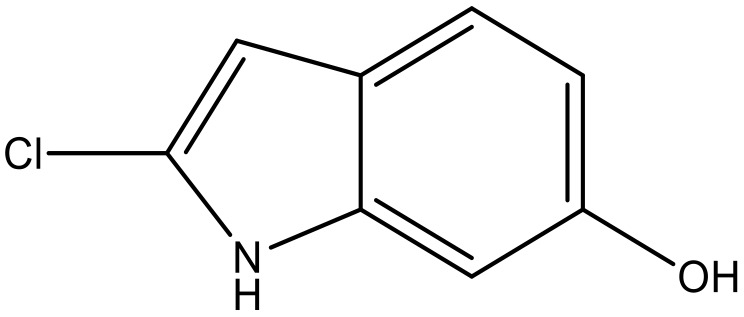	−6.959	6.664	6.612
8	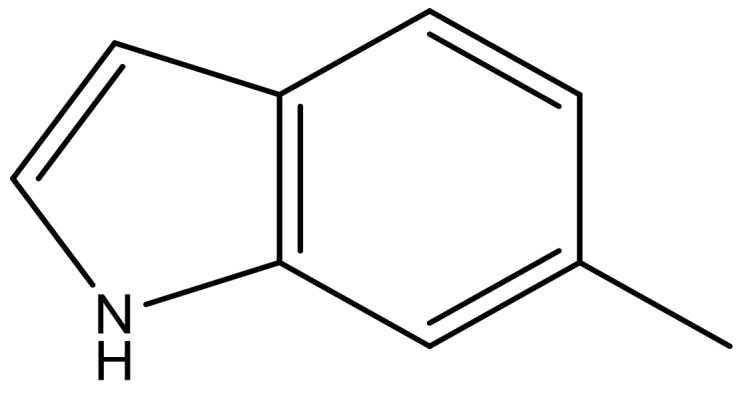	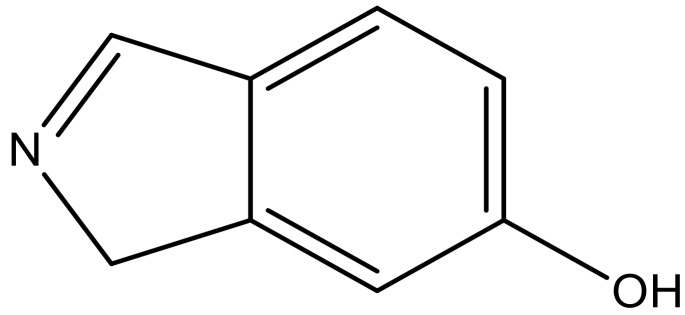	−6.421	6.367	7.058
9	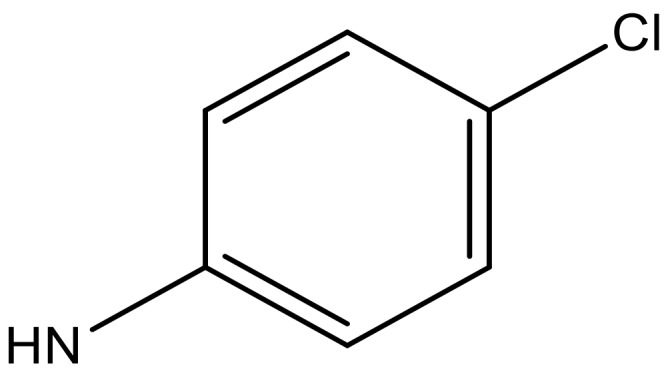	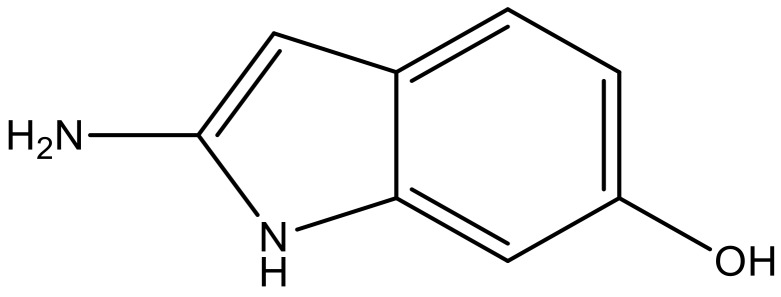	−6.429	6.347	7.035
10	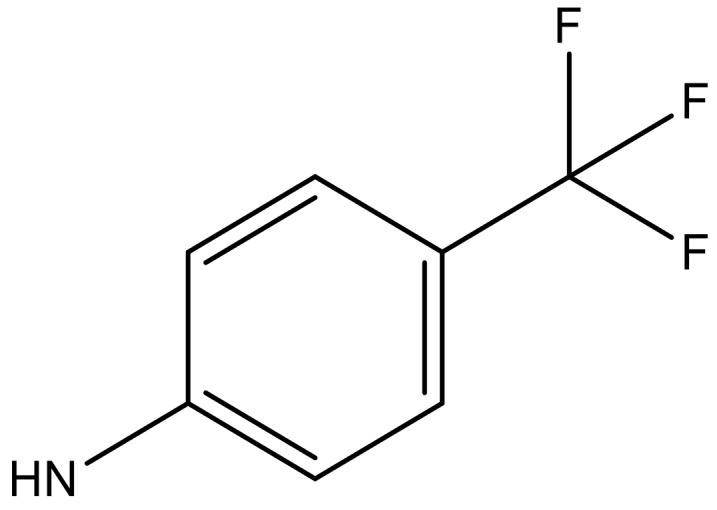	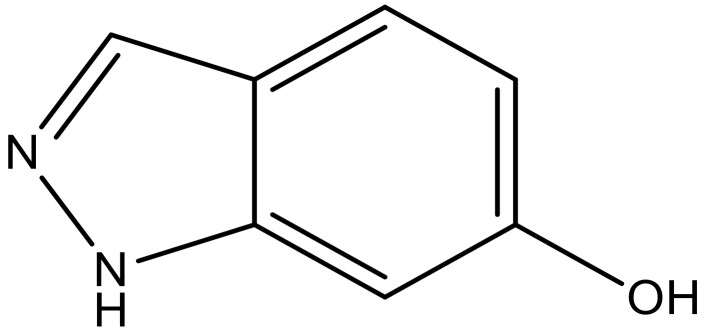	−7.352	6.302	6.123

**Table 4:** Structures of the BRD4 inhibitors and their biological activities. 
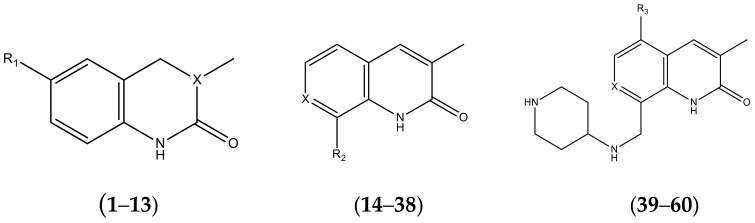

No.	R1	X	pIC_50_	No.	R1	X	pIC_50_
1	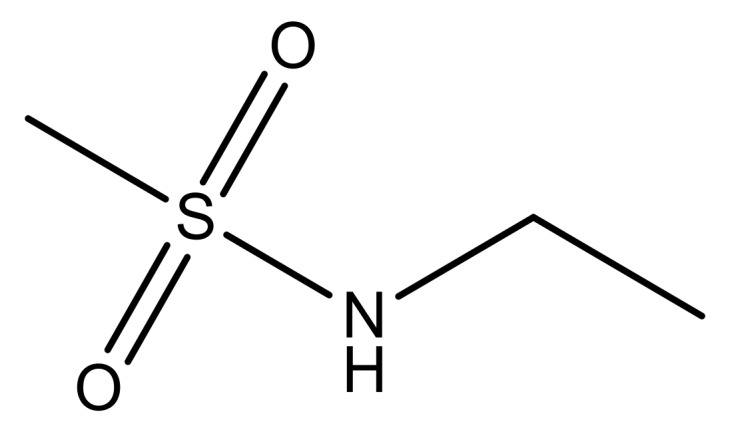	N	5.3	8	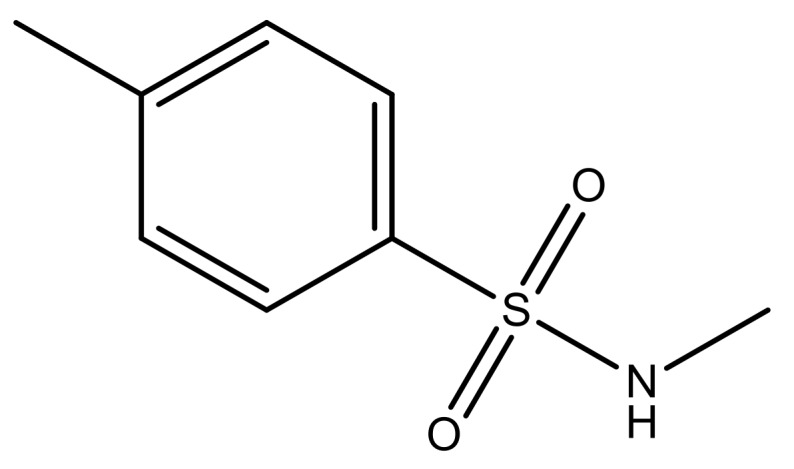	N	6.4
2	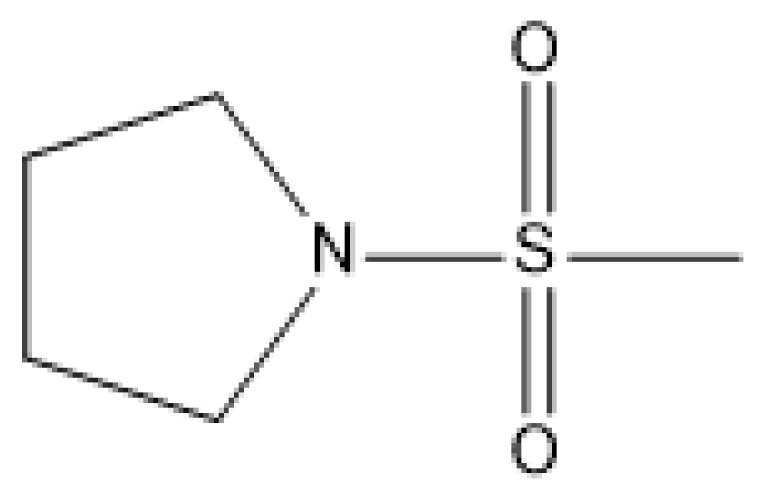	N	5.7	9	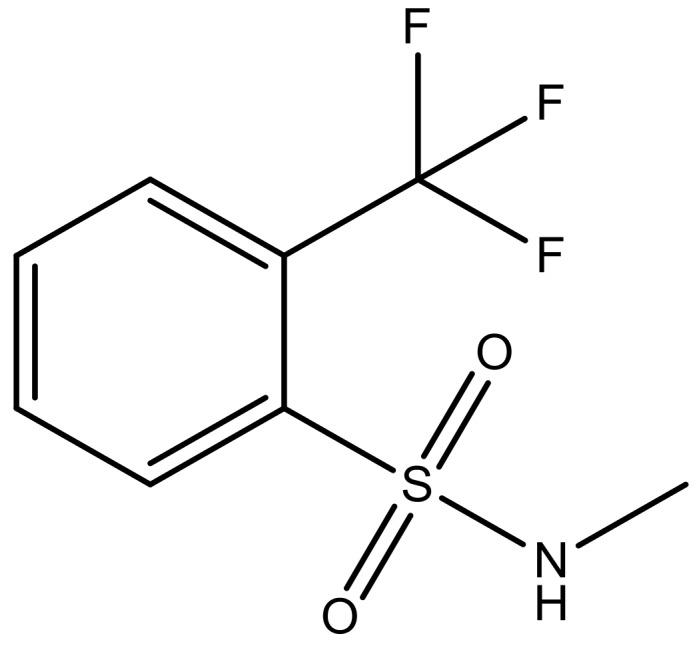	N	6.3
3	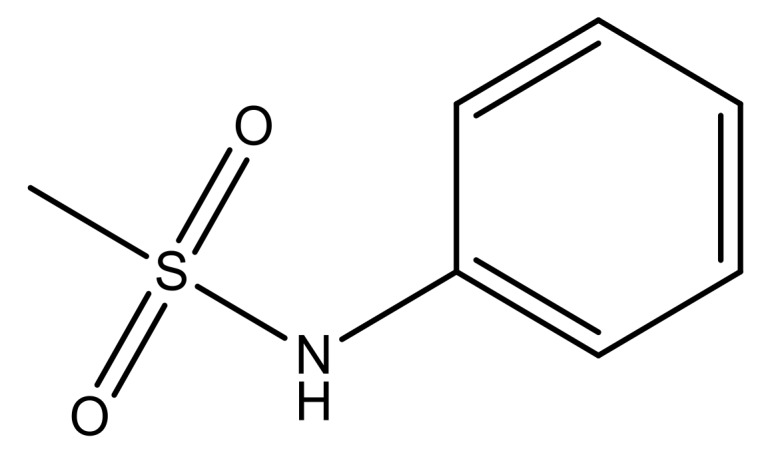	N	5.3	10	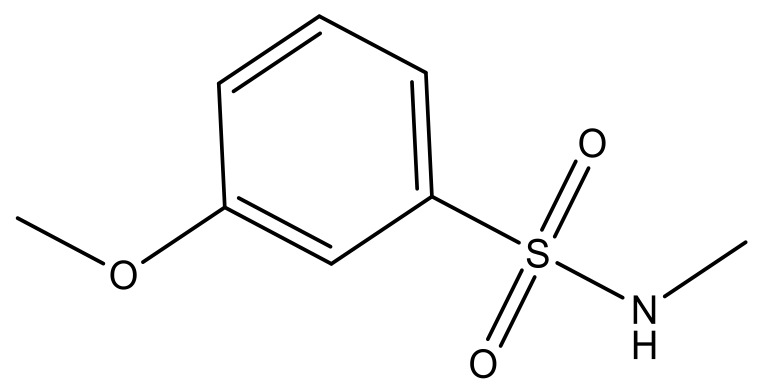	N	6.1
4	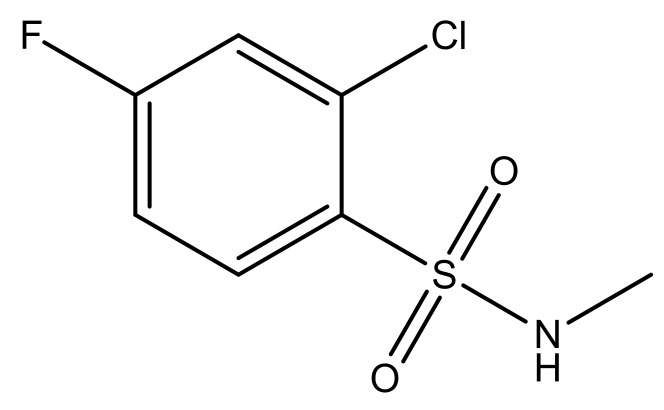	N	6.7	11	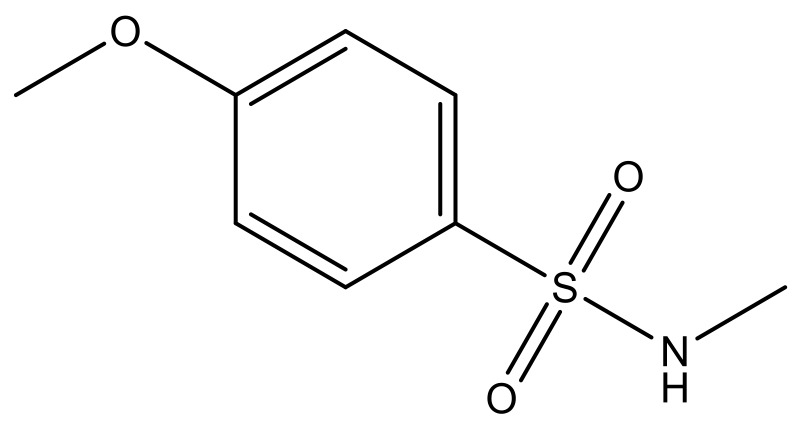	N	6.0
5	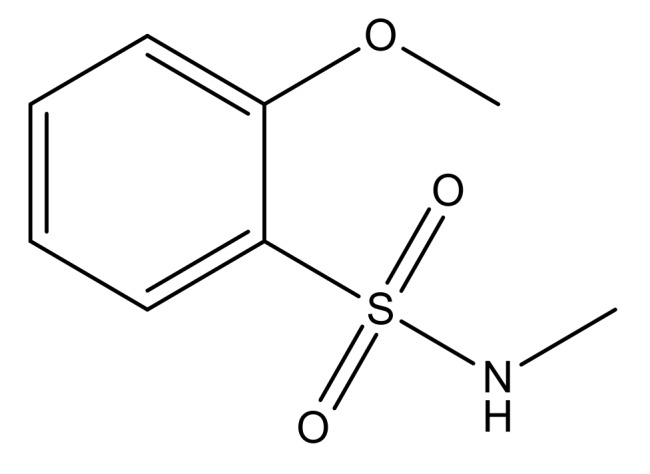	N	6.6	12	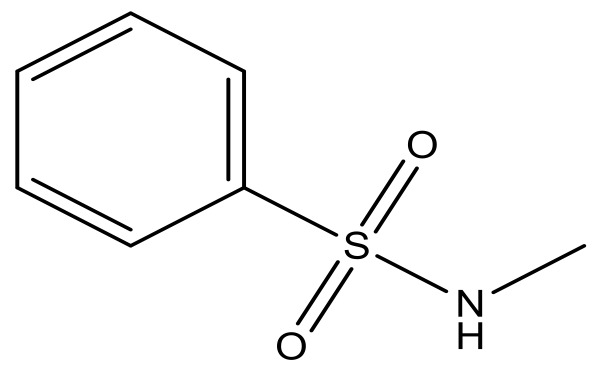	N	6.0
6	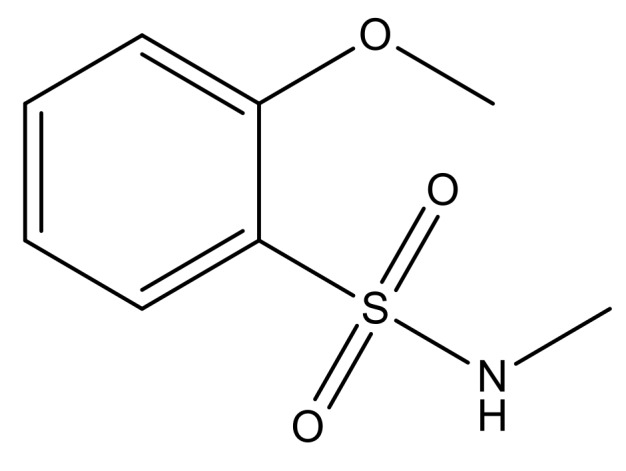	N	6.6	13	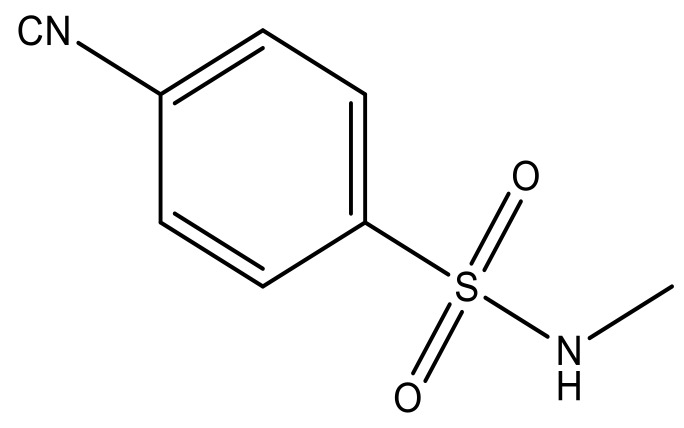	N	5.6
7*	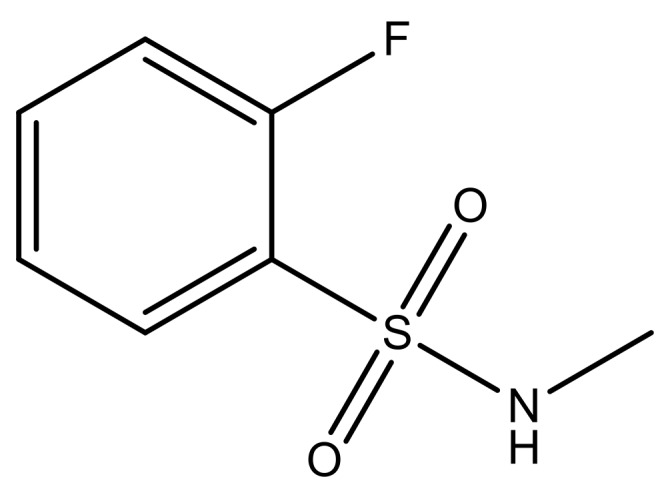	N	6.4				
**No.**	**R2**	**X**	**pIC_50_**	**No.**	**R2**	**X**	**pIC_50_**
14	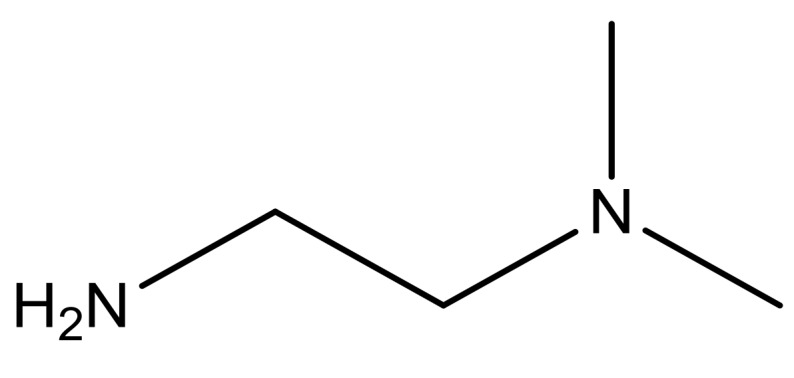		4.6	27	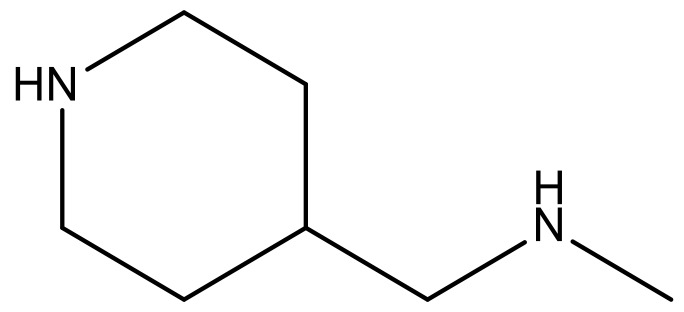	N	4.6
15	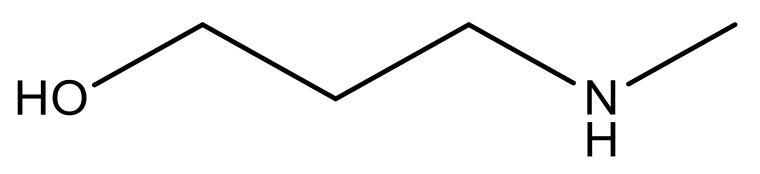		4.4	28 *	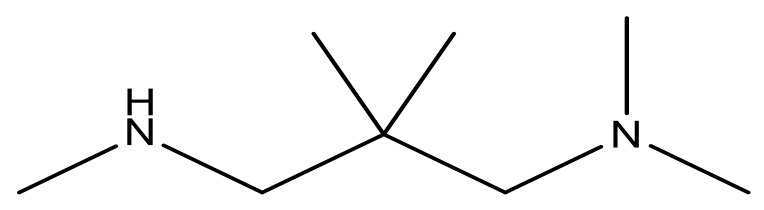	N	3.8
16*	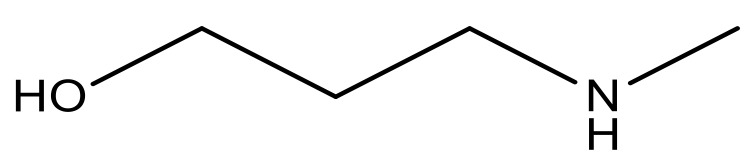		5.3	29	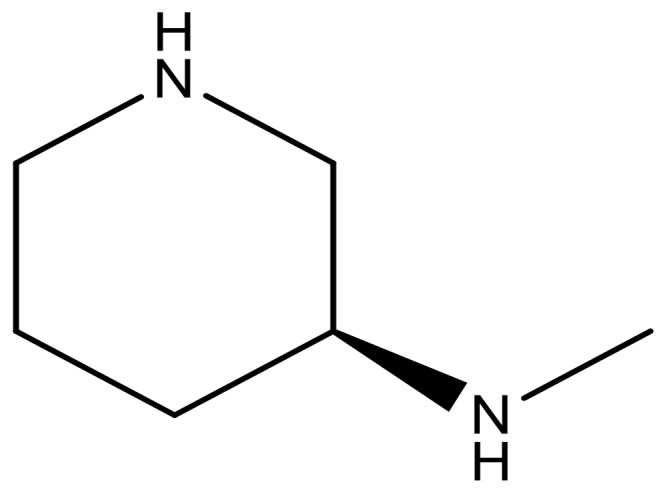	N	4.8
17	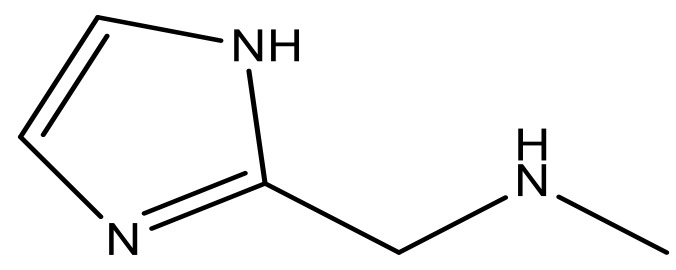		4.3	30 *	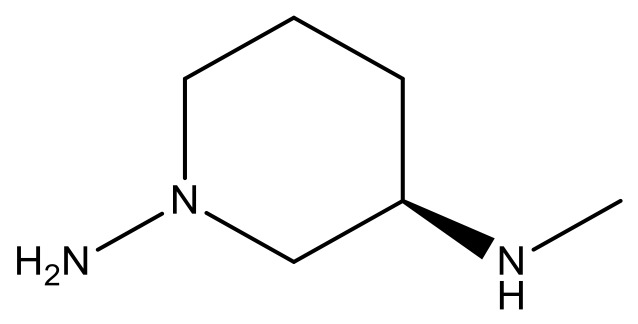	N	4.9
18	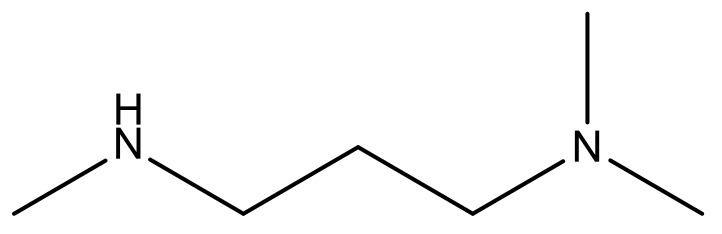	CH	5.0	31	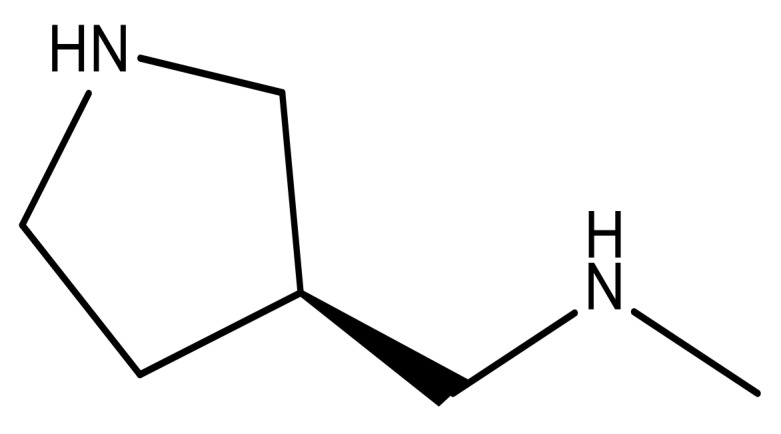	N	4.9
19	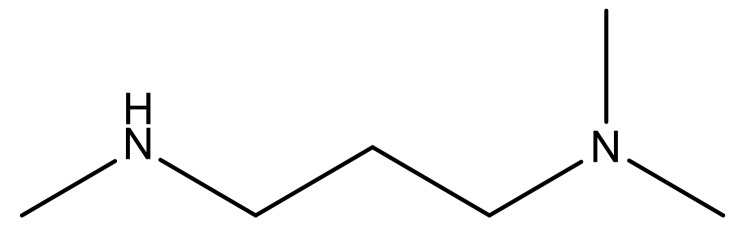	N	4.7	32	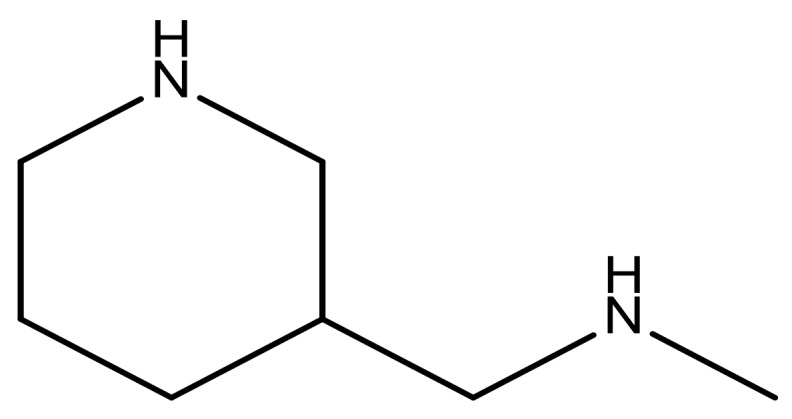	N	5.2
20	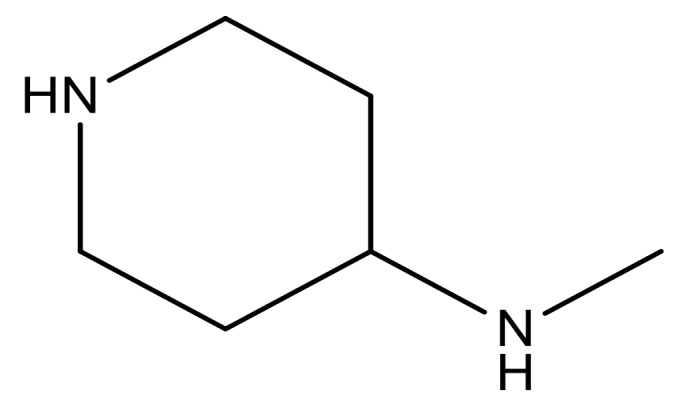	CH	5.2	33 *	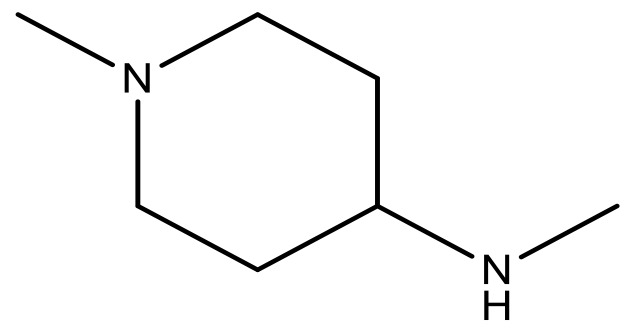	N	5.5
21	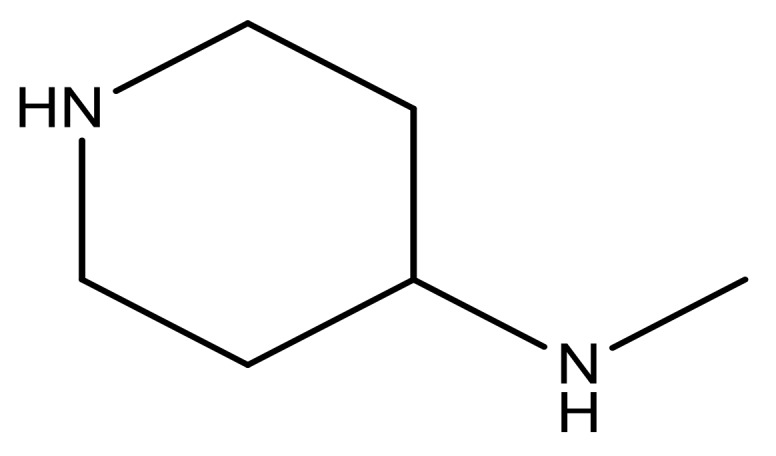	N	5.4	34	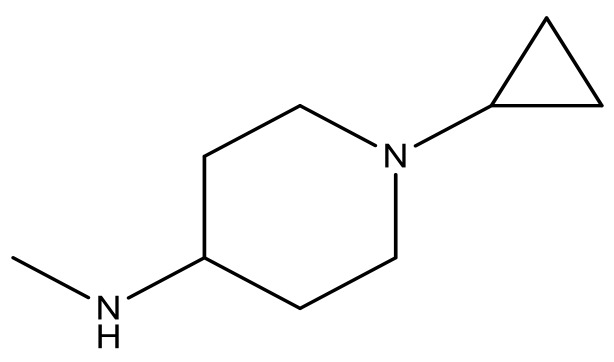	N	5.3
22*	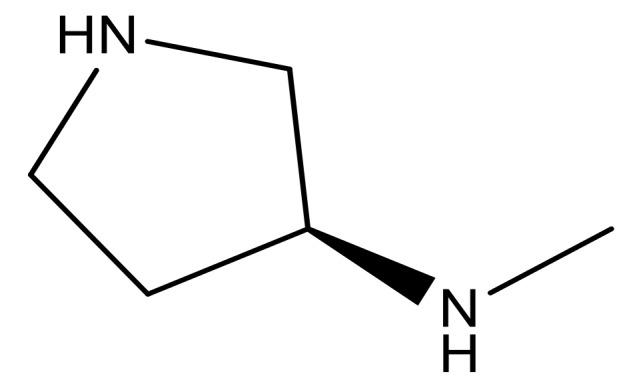	CH	5.2	35	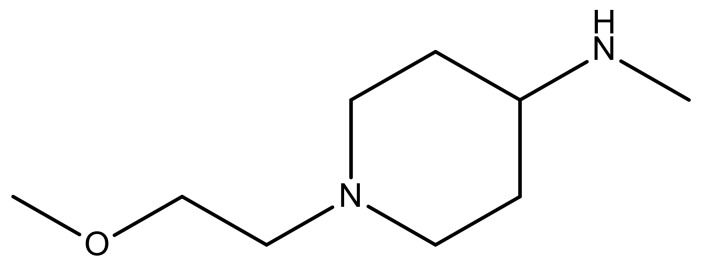	N	5.1
23	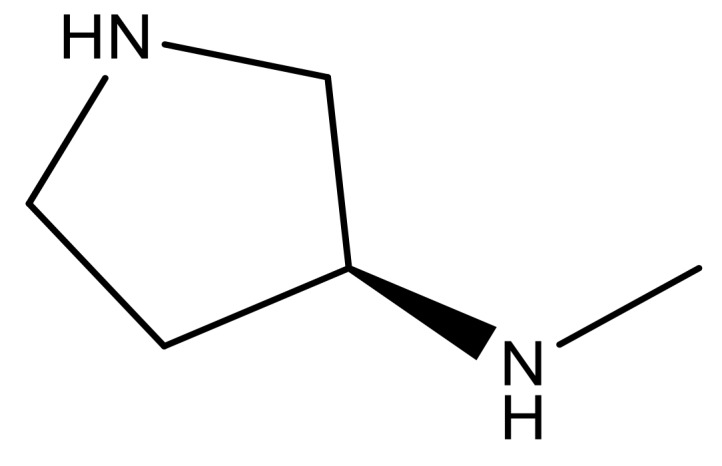	N	4.8	36	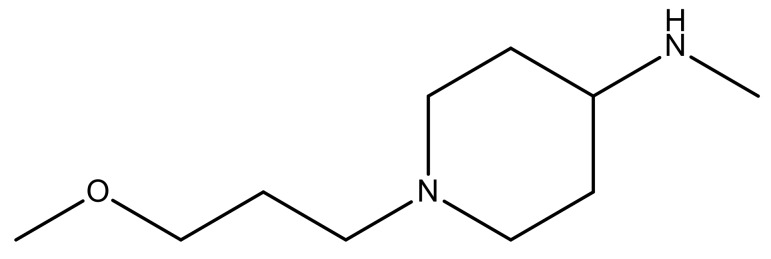	N	5.4
24	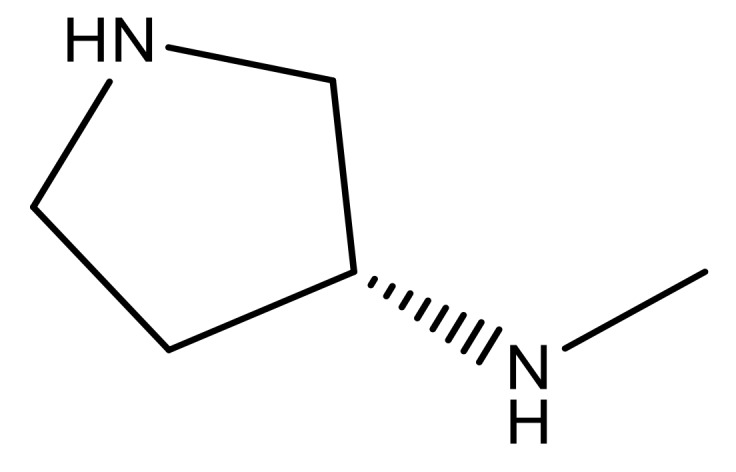	N	4.4	37	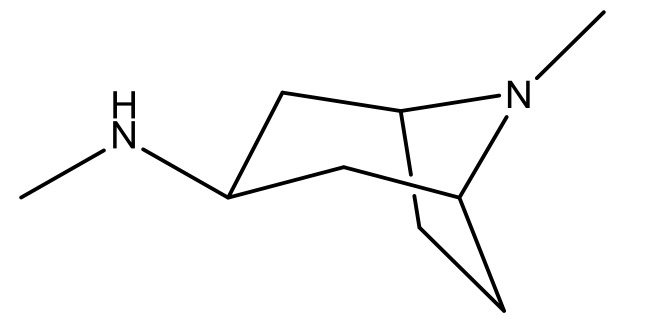	N	5.5
25	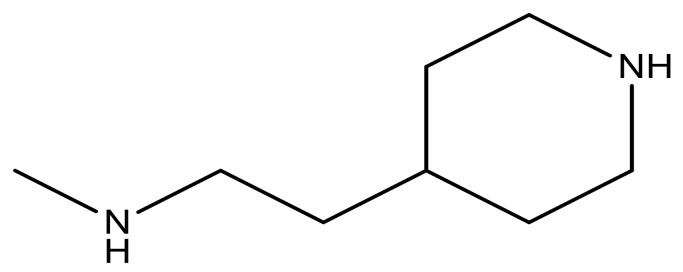	N	4.9	38	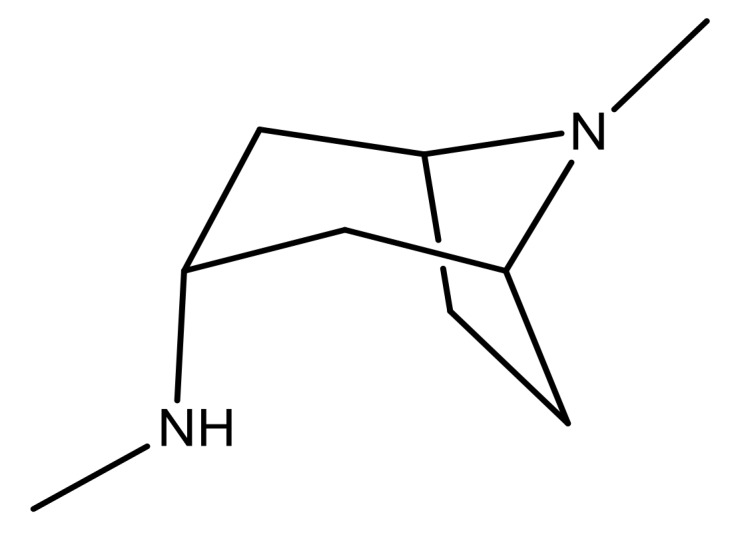	N	5.4
26	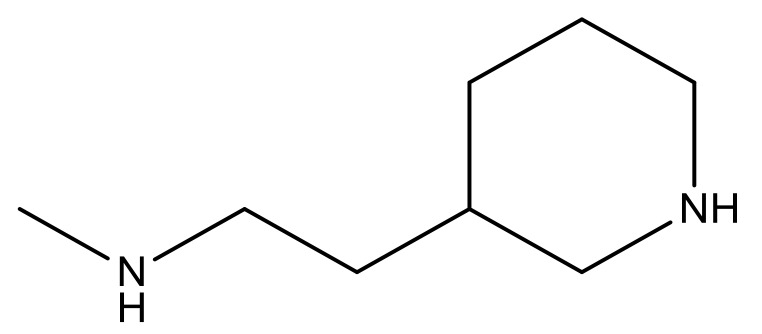	N	4.6				
**No.**	**R3**	**X**	**pIC_50_**	**No.**	**R3**	**X**	**pIC_50_**
39	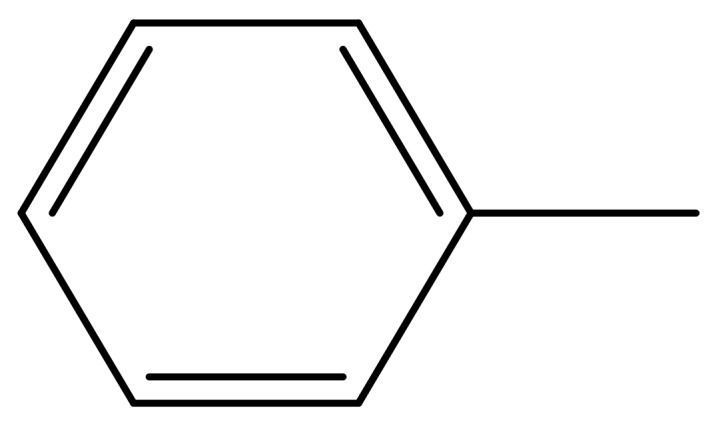	N	6.1	50 *	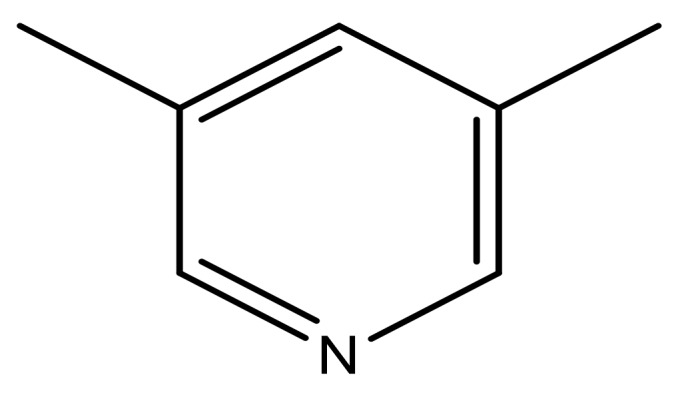	N	5.6
40	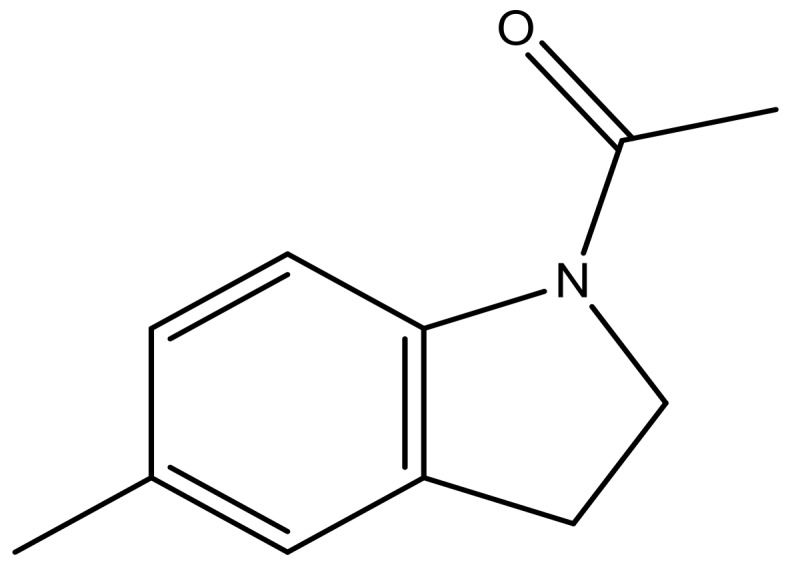	N	6.0	51	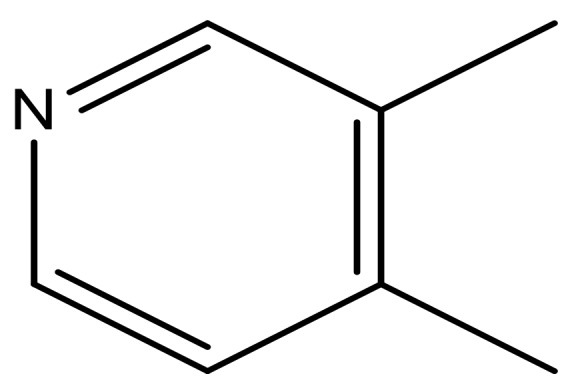	N	5.5
41	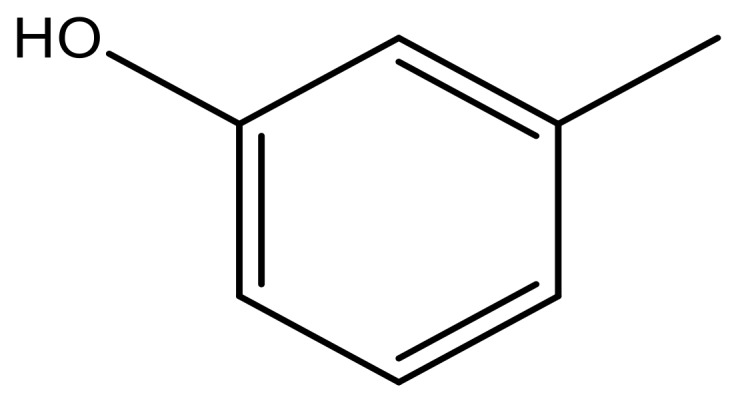	N	6.2	52	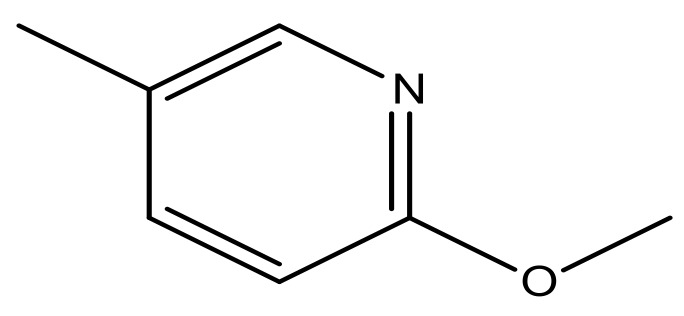	N	5.9
42	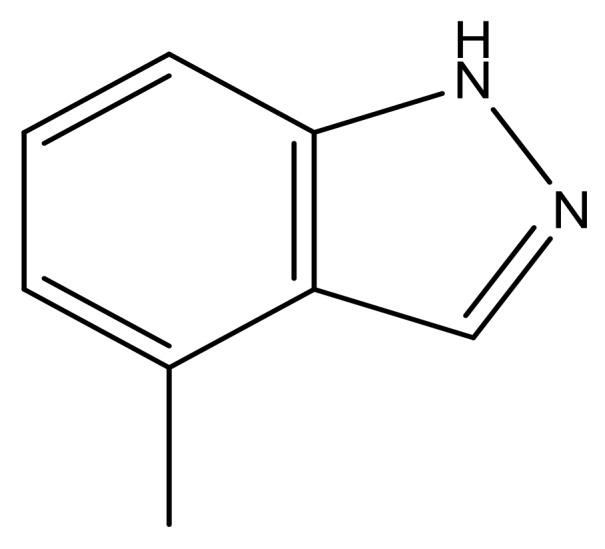	N	6.9	53	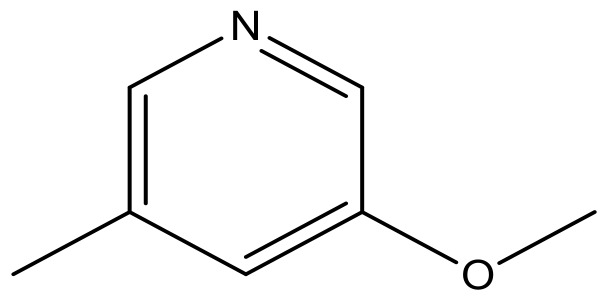	N	5.9
43	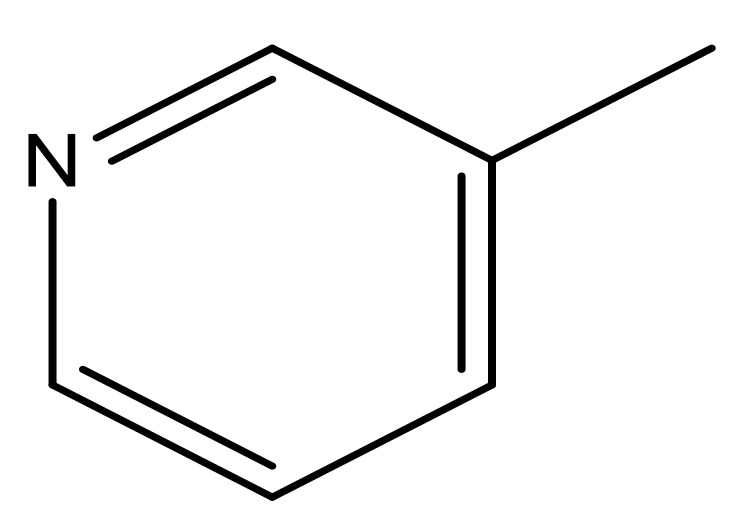	N	5.7	54	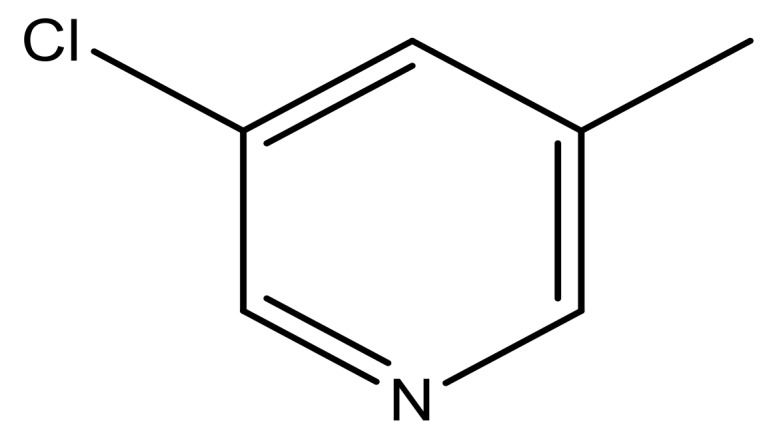	N	5.2
44	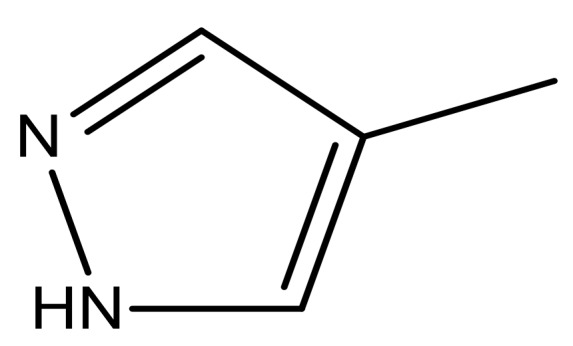	N	6.1	55 *	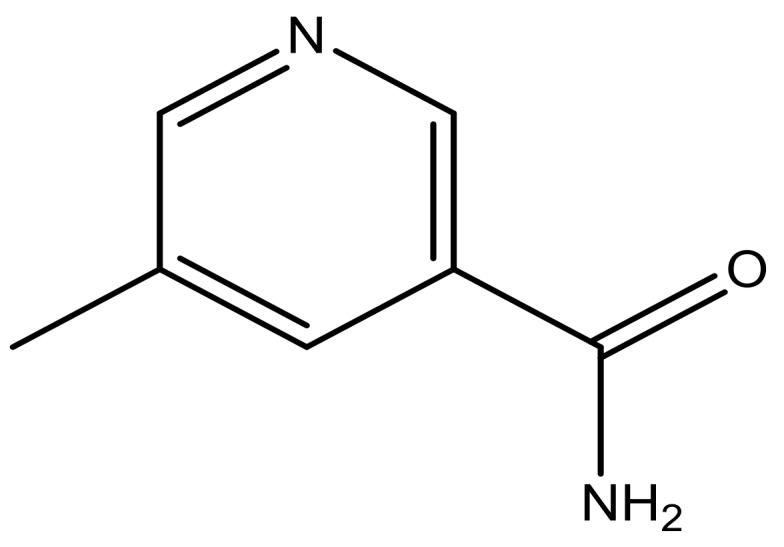	N	5.4
45	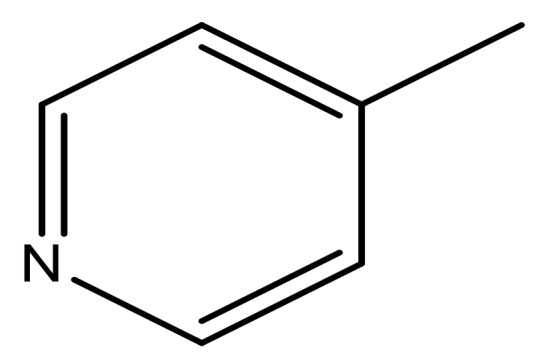	N	5.5	56	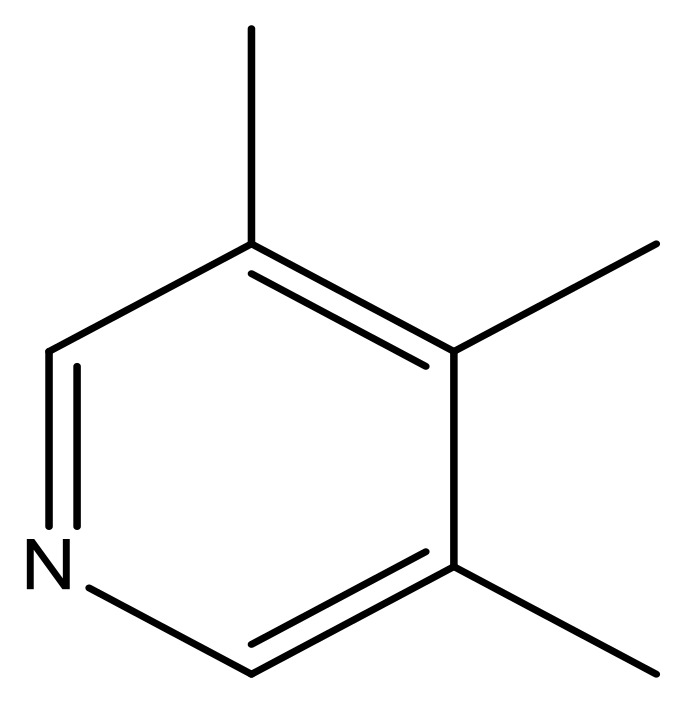	N	5.1
46 *	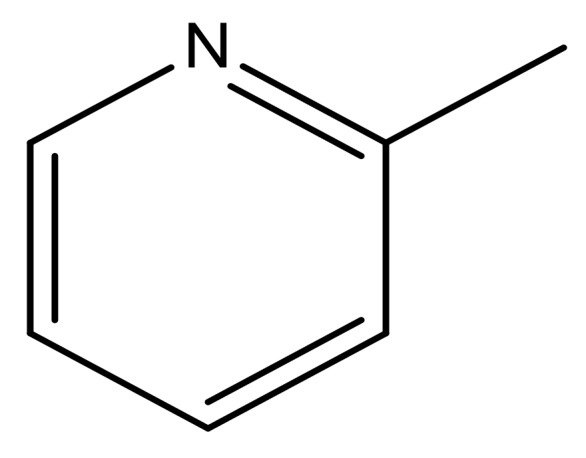	N	5.3	57	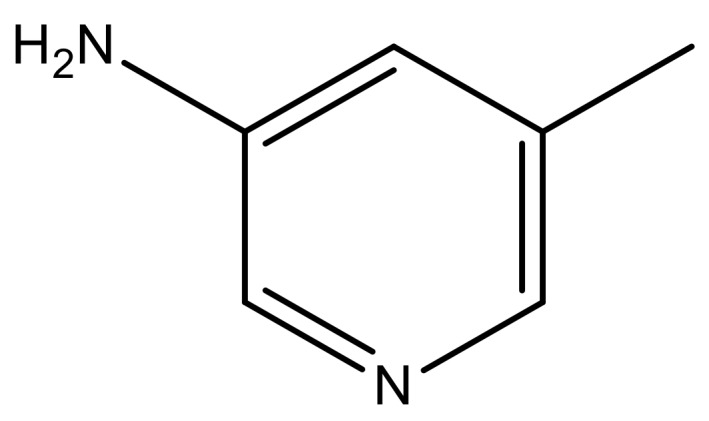	N	5.7
47	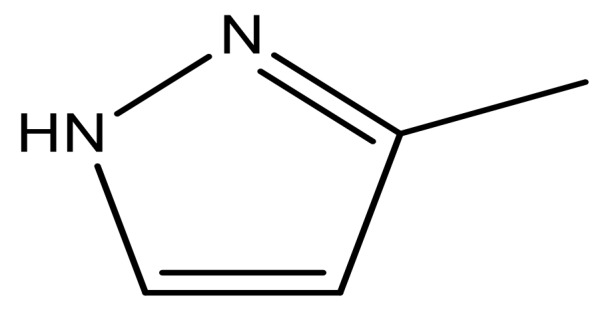	N	5.9	58	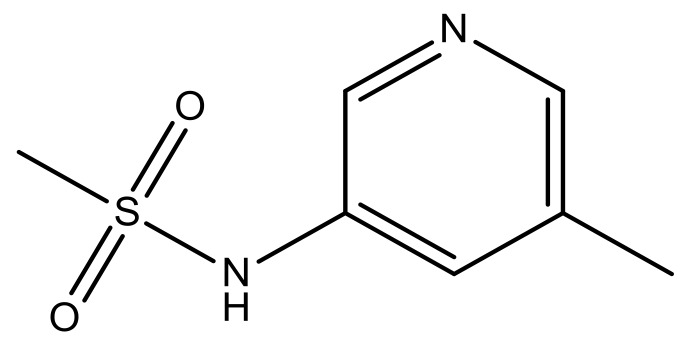	N	6.1
48 *	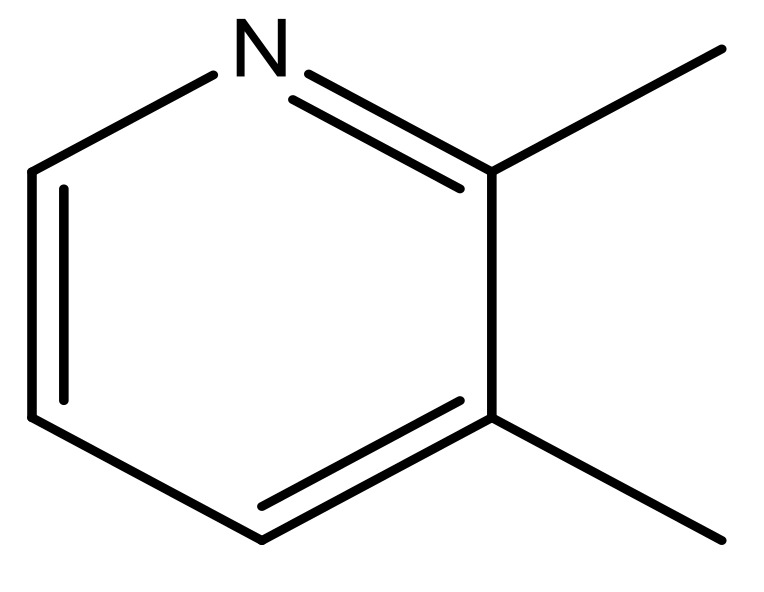	N	5.2	59	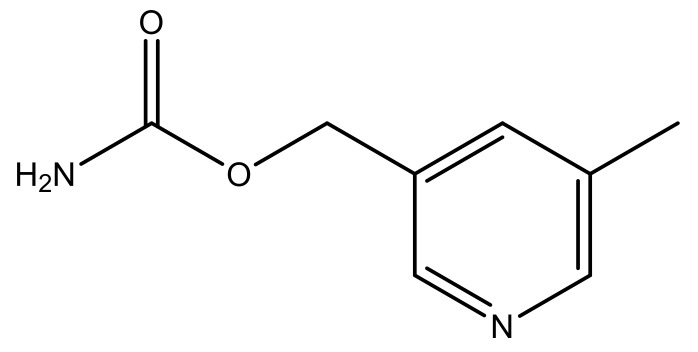	N	5.9
49	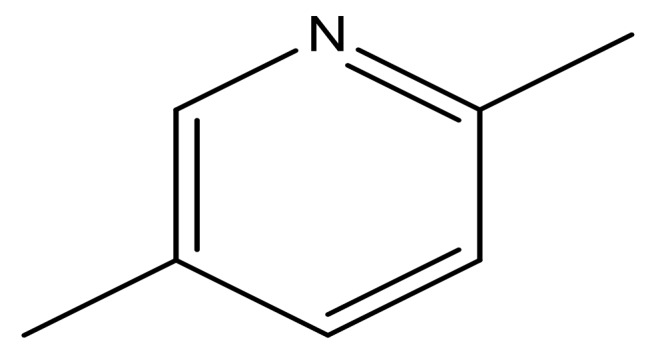	N	6.0	60	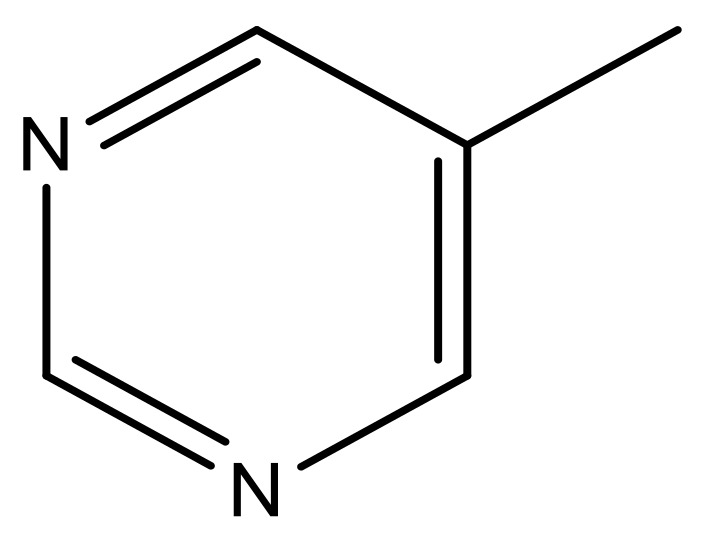	N	4.8

* Test set compounds.

## Supplementary Materials

The following are available online. Table S1: Statistical Results of Structure-Based Model (Gasteiger Huckle Charges); Table S2: Statistical Results of Structure-Based Model (Gasteiger Marsili Charges); Table S3: Statistical Results of Ligand-Based Model (Gasteiger Huckle Charges; Table S4: Statistical Results of Ligand-Based Model (Gasteiger Marsili Charges).
