# Supplementary material for: Investigations of Structural Requirements for BRD4 Inhibitors through Ligand- and Structure-Based 3D QSAR Approaches
Source: Molecules. 2018 Jun 25;23(7):1527. doi: 10.3390/molecules23071527 (PMC6099705; doi:10.3390/molecules23071527)
Supplement: Supplementary file 1 [file molecules-23-01527-s001.pdf]

Supplementary Material

Table S1: Statistical Results of Structure Based Model (Gasteiger Huckle Charges)

Gasteiger Huckle Charges	Receptor Based Model	
	50 Compounds	
Parameters	CoMFA	CoMSIA
ONC	5	6
$q^2_{(loo)}$	0.500	0.328
$r^2_{(ncv)}$	0.976	0.979
SEE	0.108	0.101
F	295.825	338.389
Pred-r^2	0.808	0.819
Steric (S)	0.545	0.154
Electrostatic (E)	0.455	0.244
Hydrophobic (H)	-	0.284
Donor (D)	-	0.147
Acceptor (A)	-	0.170
r^2_{bs}	0.983	0.986
SD_{bs}	0.006	0.005

Table S2: Statistical Results of Structure Based Model (Gasteiger Marsili Charges)

Gasteiger Marsili Charges	Receptor Based Model	
	50 Compounds	
Parameters	CoMFA	CoMSIA
ONC	6	2
$q^2_{(loo)}$	0.552	0.344
$r^2_{(ncv)}$	0.977	0.973
SEE	0.107	0.116
F	300.938	258.463
Pred-r^2	0.822	0.830
Steric (S)	0.498	0.141
Electrostatic (E)	0.502	0.288
Hydrophobic (H)	-	0.263
Donor (D)	-	0.143
Acceptor (A)	-	0.166
r^2_{bs}	0.984	0.984
SD_{bs}	0.007	0.007

<u>Table S3:</u> Statistical Results of Ligand Based Model (Gasteiger Huckle Charges)

Gasteiger Huckle Charges	Ligand Based Model	
	50 Compounds	
Parameters	CoMFA	CoMSIA
ONC	2	5
$q^2_{(loo)}$	0.404	0.414
$r^2_{(ncv)}$	0.869	0.873
SEE	0.255	0.250
F	47.378	49.416
Pred-r^2	0.774	0.761
Steric (S)	0.544	0.143
Electrostatic (E)	0.456	0.236
Hydrophobic (H)	-	0.251
Donor (D)	-	0.186
Acceptor (A)	-	0.184
r^2_{bs}	0.911	0.927
SD_{bs}	0.065	0.057

<u>Table S4:</u> Statistical Results of Ligand Based Model (Gasteiger Marsili Charges)

Gasteiger Marsili Charges	Ligand Based Model	
	50 Compounds	
Parameters	CoMFA	CoMSIA
ONC	2	2
$q^2_{(loo)}$	0.410	0.364
$r^2_{(ncv)}$	0.873	0.875
SEE	0.248	0.248
F	60.786	50.384
Pred-r^2	0.873	0.762
Steric (S)	0.495	0.130
Electrostatic (E)	0.505	0.276
Hydrophobic (H)	-	0.230
Donor (D)	-	0.175
Acceptor (A)	-	0.190
r^2_{bs}	0.919	0.928
SD_{bs}	0.055	0.065

ONC = optimal number of components; q^2 = cross-validated correlation coefficient; r^2 = determination coefficient; SEE = standard error of estimate; r^2_{ncv} = non-cross validated coefficient; F = Fischer's F-; Pred-r^2 = predictive r^2; S = steric, E = electrostatic, H = hydrophobic field; D = donor ; A = acceptor; r^2_{bs} = r^2 obtained after bootstrapping; SD_{bs} = bootstrapping standard deviation.
